# Combined antitumoral effects of pretubulysin and methotrexate

**DOI:** 10.1002/prp2.460

**Published:** 2019-01-22

**Authors:** Sarah Kern, Ines Truebenbach, Miriam Höhn, Jan Gorges, Uli Kazmaier, Stefan Zahler, Angelika M. Vollmar, Ernst Wagner

**Affiliations:** ^1^ Pharmaceutical Biotechnology Center for System‐Based Drug Research, and Center for Nanoscience (CeNS) Ludwig‐Maximilians‐Universität Munich Germany; ^2^ Institute for Organic Chemistry Saarland University Saarbrücken Germany; ^3^ Pharmaceutical Biology Center for System‐Based Drug Research Ludwig‐Maximilians‐Universität Munich Germany

**Keywords:** actin, apoptosis, cancer, cell cycle, chemotherapy, tubulin

## Abstract

Pretubulysin (PT), a potent tubulin‐binding antitumoral drug, and the well‐established antimetabolite methotrexate (MTX) were tested separately or in combination (PT+MTX) for antitumoral activity in L1210 leukemia cells or KB cervix carcinoma cells in vitro and in vivo in NMRI‐nu/nu tumor mouse models. In cultured L1210 cells, treatment with PT or MTX displays strong antitumoral effects in vitro*,* and the combination PT+MTX exceeds the effect of single drugs. PT also potently kills the MTX resistant KB cell line, without significant MTX combination effect. Cell cycle analysis reveals the expected arrest in G1/S by MTX and in G2/M by PT. In both cell lines, the PT+MTX combination induces a G2/M arrest which is stronger than the PT‐triggered G2/M arrest. PT+MTX does not change rates of apoptotic L1210 or KB cells as compared to single drug applications. Confocal laser scanning microscopy images show the microtubule disruption and nuclear fragmentation induced by PT treatment of L1210 and KB cells. MTX changes the architecture of the F‐actin skeleton. PT+MTX combines the toxic effects of both drugs. In the in vivo setting, the antitumoral activity of drugs differs from their in vitro cytotoxicity, but their combination effects are more pronounced. MTX on its own does not display significant antitumoral activity, whereas PT reduces tumor growth in both L1210 and KB in vivo models. Consistent with the cell cycle effects, MTX combined at moderate dose boosts the antitumoral effect of PT in both in vivo tumor models. Therefore, the PT+MTX combination may present a promising therapeutic approach for different types of cancer.

**Abbreviations**
CLSM: confocal laser scanning microscopy
DHFR: dihydrofolate reductase
DMSO: dimethyl sulfoxide
FCS: fetal calf serum
FITC/PI: annexin V‐fluorescein isothiocyanate/propidium iodide
FR: folate receptor
HBG: HEPES buffered glucose
IC50: half maximal inhibitory concentration
MTT: 3‐(4,5‐dimethylthiazol‐2‐yl)‐2,5‐diphenyltetrazolium bromide
MTX: methotrexate
PBS: phosphate‐buffered saline
POMP: 6‐mercaptopurine (Purinethol), vincristine (Oncovin), methotrexate, prednisone
PT: pretubulysin
RFC: reduced folate carrier

## 1 INTRODUCTION

Given their central role in the cell division process, microtubules represent major targets for chemotherapeutic drugs. Microtubule‐targeting agents exhibit highly effective anticancer properties and are used widely in the clinics. By either stabilizing or destabilizing microtubules, they lead to a disruption of the microtubule network and to G2/M arrest.^1^ However, given the development of resistances that for instance frequently occur with Vinca alkaloids, the need for new drugs of this class becomes crucial.^2^, ^3^ Tubulysins, a family of natural compounds of myxobacterial origin, are a powerful and highly effective therapeutic group. By binding to the vinca domain of β‐tubulin, tubulysins prevent tubulin polymerization which ultimately results in microtubule depletion and apoptosis of the treated cells.^4^, ^5^ Pretubulysin (PT) is a biosynthetic precursor of the tubulysins and better accessible by chemical synthesis.^6^, ^7^ Moreover, it displays remarkable antitumoral potency in the subnanomolar region.^6^, ^8^ PT not only leads to reduced tumor cell growth of different cell lines^6^ and inhibits cancer cell migration in vitro,^8^ it also shows great potential in vivo: PT inhibits tumor growth^6^, ^8^, ^9^, ^10^, ^11^ and metastasis^6^, ^12^ and also significantly reduces angiogenesis.^8^, ^10^

Antifolates, which belong to the class of antimetabolites, were among the first chemotherapeutic drugs to be investigated for the cure of metastatic cancer. As folate antagonists, they make use of the reduced folate carrier (RFC) or the folate receptor (FR) to enter the cell.^13^, ^14^, ^15^ The FR is overexpressed in many epithelial tumors and therefore plays an important role in targeted cancer therapy.^16^, ^17^ Methotrexate (MTX) is the most prominent representative of this group. After successfully entering the cell, it competitively inhibits the dihydrofolate reductase (DHFR) and by that the conversion of folic acid to dihydrofolic acid and tetrahydrofolic acid. These are required for the biosynthesis of purines, thus the de novo synthesis of DNA.^18^ However, acquired resistance to MTX represents a common problem of monotherapy approaches.^19^, ^20^, ^21^ This hurdle can possibly be overcome by combining MTX with a second antitumoral agent.

Combination therapy is an approach that combines two or more therapeutic agents in order to address several targets, possibly reduce resistance formation and increase the therapeutic efficacy while potentially decreasing dosages.^22^, ^23^ Already in 1965, a combination chemotherapy approach referred to as POMP regimen was successfully administered. Apart from MTX, it contained 6‐mercaptopurine, vincristine, and prednisone and resulted in long‐term remission in children with acute lymphocytic leukemia.^24^, ^25^

In a previous experiment, in which the novel tubulin inhibitor PT was conjugated with MTX‐containing oligomers for FR‐targeted delivery, we searched for a possible combination effect.^11^ Hence, this drug combination was chosen in this study to further boost the encouragingly high potency of PT. The combination of both drugs was analyzed in terms of cytotoxicity, apoptosis, its influence on the tumor cell cycle and the cytoskeleton. Moreover, in vivo antitumor activity of the combination approach was evaluated in a treatment experiment in two tumor mouse models. All experiments were performed in L1210 leukemia and KB cervix carcinoma cells since both are sensitive to PT and MTX. Furthermore, MTX has been well established for the treatment of leukemia.

## 2 MATERIALS AND METHODS

### 2.1 Materials

Cell culture media, antibiotics, and fetal calf serum (FCS) were obtained from Invitrogen (Karlsruhe, Germany). Flasks, dishes, and multi‐well plates were acquired from TPP (Trasadingen, Switzerland). Glucose was purchased from Merck (Darmstadt, Germany), HEPES from Biomol GmbH (Hamburg, Germany), and methotrexate from Sigma‐Aldrich (Munich, Germany, PHR1396). Pretubulysin was provided by J.G. and U.K. (Institute for Organic Chemistry, Saarland University, Saarbrücken, Germany).

### 2.2 Methods

#### 2.2.1 In vitro experiments

##### Compound preparation

For in vitro and in vivo experiments, stock solutions of PT and MTX and the combination PT+MTX were prepared at 1000‐10 000 μmol L^−1^. The powdered drugs were dissolved in 10% DMSO and 90% HBG. Further working solutions were prepared by dilution of the stock solutions with HBG. The DMSO content of the working solutions did not exceed 1%.

##### Cell culture

L1210 leukemia cells (kindly provided by Prof. Philip S. Low, Department of Chemistry, Purdue University, USA) and KB cervix carcinoma cells (ATCC; Wesel, Germany) were chosen for the following experiments. Both cell lines were grown in RPMI‐1640 medium at 37°C in 5% CO_2_. Medium was supplemented with 10% fetal calf serum (FCS), 4 mmol L^−1^ stable glutamine, 100 U mL^−1^ penicillin, and 100 μg mL^−1^ streptomycin. For cell culture experiments with KB cells, cell culture plates were coated with collagen (0.01% collagen A in HCl, Biochrom, Berlin, Germany) for 30 min at 37°C prior to seeding. Mycoplasma test was negative for both cell lines.

##### Cell viability assay (MTT)

L1210 suspension cells were seeded at a density of 5 × 10^3^ cells per well (96‐well plate) in 80 μL growth medium 4 hours prior to treatment. Twenty microliters of HBG, PT, MTX, or PT+MTX in HBG were added and plates were left to incubate for 72 hours. Ten microliters of MTT (5 mg mL^−1^) were added to each well, and incubated for 2 hours in a cell culture incubator. For cell lysis, a solution of 10% sodium dodecyl sulfate (SDS) in 0.01 mol L^−1^ hydrochloric acid (HCl) was added and incubated overnight before photometric analysis.

KB cells were seeded at a density of 2.5 × 10^3^ cells per well in 100 μL of growth medium 24 hours prior to treatment. Medium was changed 1 hour before treatment. Twenty microliters of HBG, PT, MTX, or PT+MTX in HBG were added, and plates were left to incubate for 72 hour. Ten microliters of MTT (5 mg mL^−1^) were added to each well and incubated for 2 hours in a cell culture incubator. After formazan formation, medium and MTT were removed, and cells were frozen at −80°C. After at least 30 minutes in the freezer, formazan was dissolved in 100 μL of DMSO. Absorption was measured at a wavelength of 590 nm against a reference wavelength of 630 nm using a SpectraFluor™ Plus microplate reader (Tecan, Groedig, Austria). Cell viability was calculated as percentage of absorption compared to wells treated with HBG only. All experiments were performed in quintuplicates.

##### Cell cycle analysis

L1210 cells were seeded at a density of 1 × 10^5^ cells per well (12‐well plate, 960 μL cell suspension per well) 4 hours prior to addition of 40 μL drug solution. The cells were treated with PT, MTX, and PT+MTX in HBG to a final drug concentration of 200 nmol L^−1^ PT and 600 nmol L^−1^ MTX on cells. KB cells were seeded at a density of 5 × 10^4^ cells per well. After 24 hours, medium was changed and 960 μL of fresh medium was added, 30 minutes prior to addition of 40 μL drug solution containing PT, MTX, and PT+MTX in HBG to a final drug concentration of 200 nmol L^−1^ PT and 600 nmol L^−1^ MTX on cells. Cells were incubated for 24 or 48 hours. L1210 cells were collected by centrifugation, KB cells were detached with T/E prior to collection and washed with PBS. Then, 100 μL of propidium iodide treatment solution (0.1% sodium citrate, 0.1% Triton‐X100, 50 μg mL^−1^ propidium iodide in Millipore water) was added and cells were incubated for 3 hours on ice in dark. Cells were centrifuged after adding 1 mL of PBS, resuspended in 500 μL of PBS, and measured with the CyanTM ADP flow cytometer. Data were analyzed by FlowJo 7.6.5 flow cytometric analysis software.

##### Apoptosis analysis

L1210 cells were seeded at a density of 1 × 10^5^ cells per well (12‐well plate, 960 μL cell suspension per well) 4 hours prior to treatment. The cells were treated with 40 μL drug solution containing PT, MTX, and PT+MTX in HBG to a final drug concentration of 200 nmol L^−1^ PT and 600 nmol L^−1^ MTX on cells. KB cells were seeded at a density of 5 × 10^4^ cells per well (12‐well plate, 1 mL cell suspension per well). After 24 hours, medium was changed and 960 μL of fresh medium was added, 30 minutes prior to addition of 40 μL drug solution containing PT, MTX, and PT+MTX in HBG to a final drug concentration of 200 nmol L^−1^ PT and 600 nmol L^−1^ MTX on cells. The cells were incubated for 24, 48, or 72 hours. L1210 cells were collected by centrifugation; KB cells were detached using T/E prior to collection. After a PBS wash, apoptosis was detected with an Annexin V‐FITC/PI assay (BioVision) by Cyan ADP flow cytometry. Data were analyzed by FlowJo 7.6.5 flow cytometric analysis software. Cells in different apoptotic stages were visualized with the dyes annexin V‐fluorescein isothiocyanate (FITC)/propidium iodide (PI). Annexin V has a high affinity for membrane phosphatidylserine (PS), thus FITC‐labeled annexin V can be used for the detection of outer membrane translocated PS in apoptotic cells. PI can intercalate in the DNA. It can reach the DNA as soon as the cell membrane has started to disintegrate. No dye can bind to healthy cells, they are therefore Annexin V‐FITC–/PI– (Q4). When cells start to undergo apoptosis, annexin V binds PS. PI, however, cannot yet reach the DNA. Cells are Annexin V‐FITC+/PI– (Q3). In late apoptosis or necrosis, cells are Annexin V‐FITC+/PI+ (Q2). When the cell membrane is very badly deformed, Annexin V cannot bind PS anymore even though PI can stain the DNA (Annexin V‐FITC–/PI+, Q1).

##### Confocal laser scanning microscopy

L1210 cells were seeded at a density of 1 × 10^5^ cells per well (12‐well plate, 960 μL cell suspension per well) 4 hours prior to treatment. The cells were treated with 40 μL drug solution containing PT, MTX, and PT+MTX in HBG to a final drug concentration of 200 nmol L^−1^ PT and 600 nmol L^−1^ MTX on cells. After 24, 48, or 72 hours incubation time, cells were collected by centrifugation and washed with PBS. KB cells were seeded into 8‐well μL slides at a density of 1 × 10^2^ in 300 μL of growth medium. After the respective incubation time, medium was removed and cells were washed with PBS. L1210 and KB cells were then extracted in Microtubule Stabilizing Buffer (80 mmol L^−1^ PIPES pH 6.8, 1 mmol L^−1^ MgCl_2_, 5 mmol L^−1^ EGTA‐K, and 0.5% Triton X‐100) for 30 seconds to remove monomeric and dimeric tubulin subunits. Glutaric aldehyde was added to a final concentration of 0.5% and cells were fixed for 10 minutes. A 0.1% solution of NaBH_4_ in PBS was used for subsequent quenching of unreacted glutaric aldehyde (7 minutes). Next, cells were washed with PBS: L1210 cells were collected by centrifugation before washing; KB cells were washed in the chamber slides. To block unspecific binding sites, cells were incubated with AbDil solution (TBS‐0.1% Triton X‐100, 2% BSA, 0.1% azide) for 10 minutes. The cells were incubated with the primary α‐tubulin antibody (Sigma‐Aldrich, T9026) in AbDil solution for 45 minutes. After a TBS wash, the secondary antibody (Alexa Fluor 488) was added and left on the cells for another 45 minutes. Cells were washed with TBS and incubated with a solution of DAPI (nucleus staining reagent) and phalloidin‐rhodamine (F‐actin staining reagent) in AbDil for 15 minutes. The staining solution was removed, cells were washed with TBS and 100 μL of AbDil solution was added to the KB cells in the chamber slides, which were then used for microscopy. L1210 cells were centrifuged and washed with TBS after the last staining step. TBS was removed and the cells were resuspended in 20 μL of mounting medium (Roti^®^‐Mount FluorCare, Carl Roth). Five microliters of the viscous cell suspension were added to a microscope slide, the cover slip was carefully placed on top of the drop. The cover slip was sealed with nail polish. After drying, the prepared microscope slides were used for microscopy.

#### 2.2.2 In vivo experiments

##### Murine leukemia tumor model

L1210 cells (0.5 × 10^6^ cells in 150 μL PBS) were injected subcutaneously into the left flank of female 6‐week‐old mice, RJ: NMRI‐nu (nu/nu) (Janvier, Le‐Genest‐St‐Isle, France) after a minimum of 7 days of acclimation time prior to experiments. Mice were housed in isolated ventilated cages under specific pathogen‐free conditions with a 12 hours day/night interval and food and water ad libitum. After tumor cell inoculation, weight and general well‐being were monitored continuously. Tumor size was measured with a caliper and determined by formula *a* × *b* ^2^/2 (*a* = longest side of the tumor; *b* = widest side vertical to *a*) as stated by Xu et al.^26^ All animal experiments were performed according to the guidelines of the German law for the protection of animal life and were approved by the local animal ethics committee.

##### Human cervix carcinoma xenograft mouse model

KB cells (5 × 10^6^ cells in 150 μL PBS) were injected subcutaneously into the left flank of female 6‐week‐old mice, RJ: NMRI‐nu (nu/nu) (Janvier, Le‐Genest‐St‐Isle, France) after a minimum of 7 days of acclimation time prior to experiments. Experiments were otherwise performed under equal conditions like in L1210 experiment.

##### PT+MTX combination treatment experiment in L1210 tumor model

Three days after tumor cell inoculation, animals were randomly divided into four groups (n = 4). Intravenous treatments were performed eight times (on days 3, 5, 7, 10, 12, 14, 17, and 19). A contemporary treatment approach was chosen over sequential applications for reasons of animal welfare. Due to the two‐to‐three‐day‐rhythm of intravenous injections and aggressive tumor growth, each drug could be injected for up to eight times instead of four times each. Animals were injected via tail vein injection with 250 μL of PT (2 mg kg^−1^), MTX (5 mg kg^−1^), and the corresponding combination of PT with MTX or HBG buffer control. Mice were sacrificed by cervical dislocation on day 13 or day 14 in case of MTX‐treated mice, and on day 14 or later in case of HBG‐treated mice. Animals of all other groups were sacrificed at later time points once their tumor reached 1500 mm^3^ or in case of severely affected well‐being (eg, continuous weight loss, apathy, visibly enlarged lymph nodes or spleen) for reasons of animal welfare.

##### PT+MTX combination treatment experiment in KB tumor model

Intravenous treatments were started for each animal individually when tumors reached 200‐250 mm^3^ to provide reliable and homogenous tumor growth. Animals were randomly divided into four groups (n = 6 for HBG and MTX, n = 7 for PT and PT+MTX), and injections were performed eight times (three times per week). Animals were injected via tail vein injection with 250 μL of PT (2 mg kg^−1^), MTX (5 mg kg^−1^), and the corresponding combination of PT with MTX or HBG buffer control. Mice were sacrificed by cervical dislocation once their tumor reached 1500 mm^3^ or in case of severely affected well‐being (eg, continuous weight loss, apathy, visibly enlarged lymph nodes or spleen) for reasons of animal welfare.

#### 2.2.3 Statistical analysis

Results are expressed as mean + SD if not indicated elsewise. Statistical analysis was performed with unpaired student's *t* test using GraphPad Prism™ and *P* < 0.05 were considered as significant (*P < 0.05; **P < 0.01; ***P < 0.001; ****P < 0.0001; ns = no significance).

## 3 RESULTS

### 3.1 In vitro antitumoral activity of PT, MTX or PT+MTX

**Figure 1:**
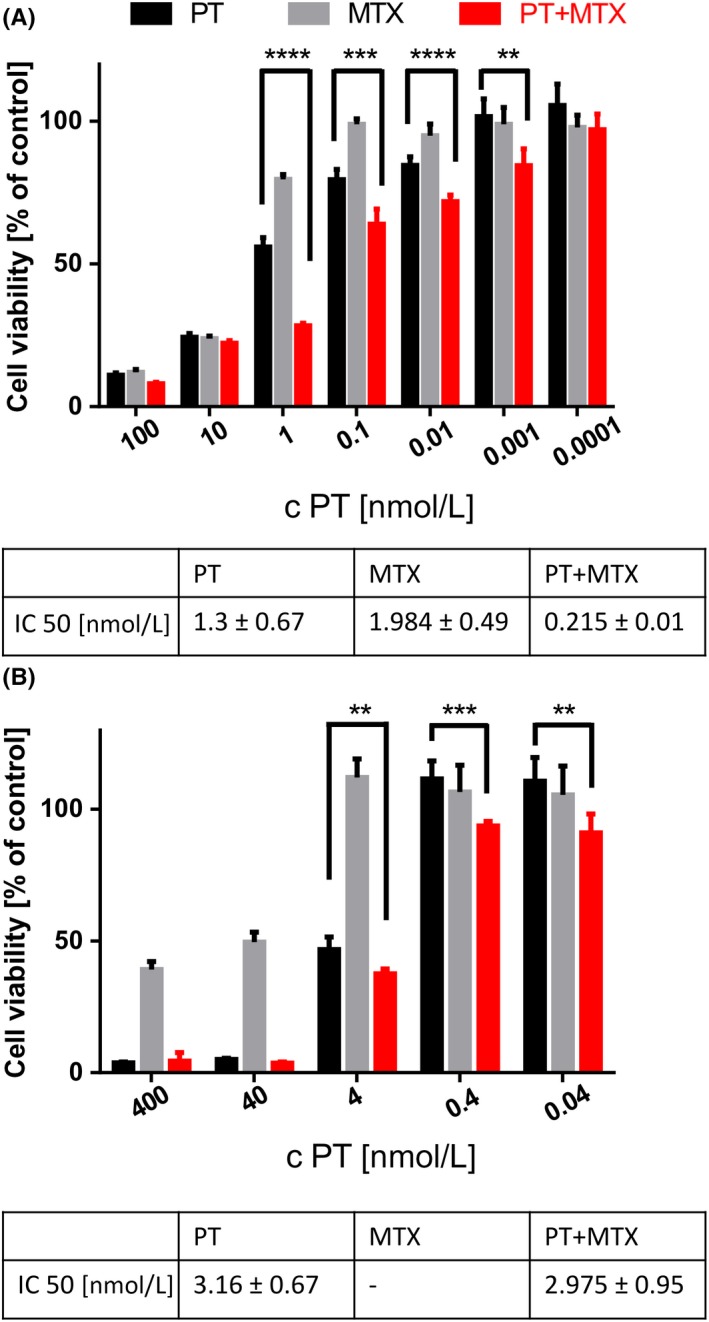
Combination effect of pretubulysin (PT) and methotrexate (MTX) on cultured L1210 cells but not KB cells. Cell viability and IC50 values of drug‐treated (A) L1210 cells and (B) KB cells. Cell viability was measured with an MTT assay after 72 hours treatment and is presented as the mean + SD (n = 5) in % relative to buffer (HEPES buffered glucose) treated cells. c (nmol L^−1^) refers to the concentration of PT, the concentration is 3‐fold higher for MTX, due to the 1:3 molar drug ratio (**P < 0.01; ***P < 0.001; ****P < 0.0001)

L1210 and KB cells were treated with PT and MTX for 72 hours at a set drug molar ratio of 1 to 3, and cell viability of drug‐treated cells was determined by MTT assay (Figure 1). In case of L1210 cells (Figure 1A) both the single drugs as well as their combination induce strong effects already at low nanomolar concentrations. The IC50 values of the single drugs in the 96‐well format are around 1 nmol L^−1^ (PT: 1.3 ± 0.067; MTX: 1.984 ± 0.49; PT+MTX: 0.215 ± 0.01), and a beneficial effect of PT+MTX over PT and MTX alone is visible. The combination effect is especially predominant at a concentration of 1 nmol L^−1^ of PT and 3 nmol L^−1^ MTX, and can also be seen when comparing the IC50 values.

KB cells (Figure 1B) are partly resistant to MTX, with a minimum cell viability of 40% remaining at high MTX concentrations. PT alone exhibits strong antitumoral effects on KB cells, with an IC50 in the low nanomolar region. The combination formulation is similarly potent as the single drug PT, as can be seen for the IC50 values in Figure 1B. At doses below 40 nmol L^−1^ of PT, the combination PT+MTX is significantly more potent than PT alone. No significant combination effect is visible at the higher drug ratios 5:1 and 10:1 (see Figure S1).

### 3.2 The effect of PT, MTX, or PT+MTX treatment on tumor cell cycle

**Figure 2:**
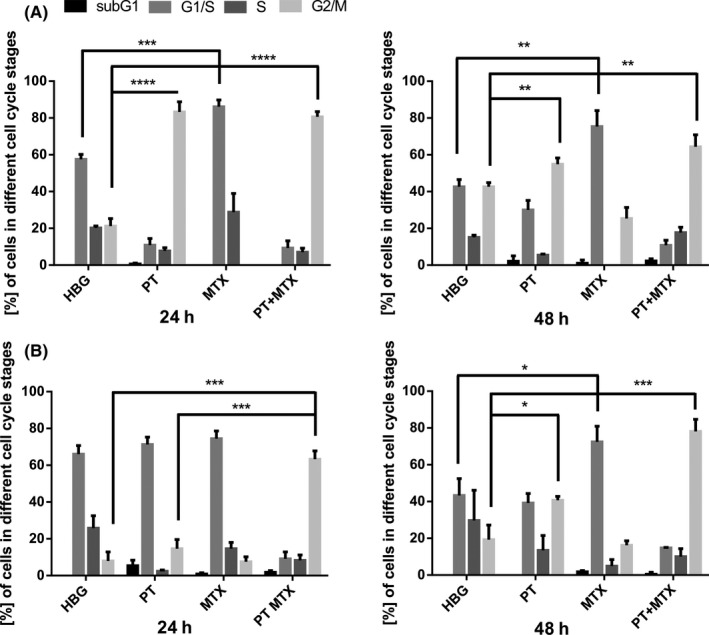
Cell cycle analysis of drug‐treated cells. (A) L1210 cells and (B) KB cells were treated with HEPES buffered glucose (HBG) buffer control, 200 nmol L^−1^ pretubulysin (PT), 600 nmol L^−1^ methotrexate (MTX), or the combination PT + MTX (200 + 600 nmol L^−1^). Cells were incubated for 24 hours, respectively 48 hours. Cells were stained with propidium iodide and analyzed by flow cytometry. Treatments were performed in triplicates (mean + SD; n = 3; *P < 0.05; **P < 0.01; ***P < 0.001; ****P < 0.0001)

L1210 and KB cells were treated with HBG, PT, MTX, or PT+MTX and left to incubate for 24 hours or 48 hours. Time points and drug concentrations were adjusted to the 12‐well plate culture conditions. Figure S2 shows cell viabilities under these conditions as determined by MTT assay. Cells were stained with the DNA intercalating dye propidium iodide and measured by flow cytometry (Figure 2). After 24 hours treatment of L1210 cells (Figure 2A), PT induces the expected strong G2/M arrest (83% arrest in G2/M), whereas MTX induces a strong G1/S arrest (86% in G1). With regard to PT+MTX co‐treatment, the pattern at 24 hours (81% arrest in G2/M) equals treatment with only PT. Interestingly, after 48 hours, the G2/M effect of PT‐treated cells is reduced (55% G2/M, 30% G1), whereas MTX still induces a strong 75% G1/S arrest. In contrast, no comparable G1/S arrest is found in the PT+MTX combination group, but a stronger G2/M arrest of cells (64% G2/M, only 11% G1) is seen when compared to the single drug PT. In sum, in the combination group, the G2/M effect of PT seems to be predominant, and the effect is even supported by MTX co‐treatment.

For KB cells (Figure 2B), changes in cell cycle are in general delayed as compared with the faster growing L1210 cells. No significant alterations can be noted after 24 hours incubation. With time, PT treatment starts to build up some G2/M arrest (41% G2/M at 48 hours), MTX‐treated cells are largely in G1 phase. In sharp contrast to the single drug treatments, the PT+MTX combination induces a strong G2/M arrest already after 24 hours, and much stronger (78% G2/M) also after 48 hours than PT alone.

### 3.3 Induction of apoptosis by PT, MTX, or PT+MTX

**Figure 3:**
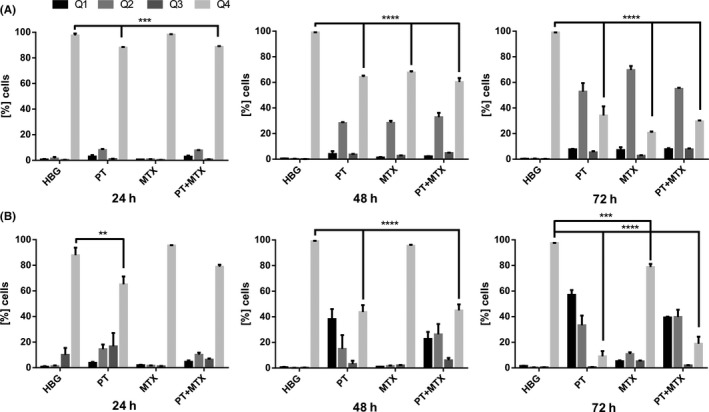
Apoptosis in drug‐treated L1210 cells (A) or KB cells (B). Cells were treated with control buffer HEPES buffered glucose (HBG), pretubulysin (PT), methotrexate (MTX), or PT + MTX and incubated for 24, 48, and 72 hours, respectively. Cells were stained with annexin V‐FITC and propidium iodide and analyzed by flow cytometry. Treatments were performed in triplicates (mean + SD; n = 3; **P < 0.01; ***P < 0.001; ****P < 0.0001). Q1: Annexin V‐FITC–/PI+; Q2: Annexin V‐FITC+/PI+; Q3: Annexin V‐FITC+/PI–; Q4: Annexin V‐FITC–/PI–

The effect of drug treatment on apoptosis of L1210 (Figure 3A and Figure S3) and KB cells (Figure 3B and Figure S4) was monitored with an annexin V‐fluorescein isothiocyanate (FITC) / propidium iodide (PI) assay. L1210 and KB cells were treated with HBG control buffer, PT, MTX, or PT+MTX and left to incubate for 24, 48, or 72 hours. Cells were collected, stained with annexin V‐FITC and PI and analyzed by flow cytometry. For L1210 cells (Figure 3A and Figure S3), HBG and MTX treatment for 24 hours did not trigger any signs of apoptosis (Q4), whereas PT and PT+MTX‐induced apoptosis in 10% of cells (Q1‐Q3). After 48 hours, 30% of MTX‐treated and 30%‐40% of PT or PT+MTX‐treated L1210 cells were apoptotic. After 72 hours incubation time, 80% of MTX‐treated cells and 70% of PT or PT+MTX‐treated cells show signs of apoptosis. Combining PT and MTX does not increase the number of apoptotic cells in comparison to the single drugs. Apoptotic cells are mainly in the Q2 quadrant, reflecting that cells are in the late apoptotic or necrotic phase.

After treatment of KB cells (Figure 3B and Figure S4) for 24 hours, MTX‐treated cells did not show apoptotic signs (Q4), whereas PT and PT+MTX‐treated cells were 20% or 30% apoptotic (Q1‐Q3). The number of PT or PT+MTX‐treated apoptotic cells steadily increases over the time course of the experiment, with only around 15% of healthy cells remaining. In contrast to L1210 cells, the majority of apoptotic KB cells were FITC–/PI+ (Q1), indicating that the cell membrane of KB cells was too heavily destroyed to bind annexin V. Consistent with MTT and cell cycle data, the MTX resistance of KB cells was also noted in the apoptosis analysis; only 20% of MTX‐treated cells had undergone apoptosis after 72 hours treatment. Like for L1210 cells, the combination PT+MTX did not enhance the number of apoptotic KB cells over the single drug treatments.

### 3.4 Confocal laser scanning microscopy of drug‐treated cells

**Figure 4:**
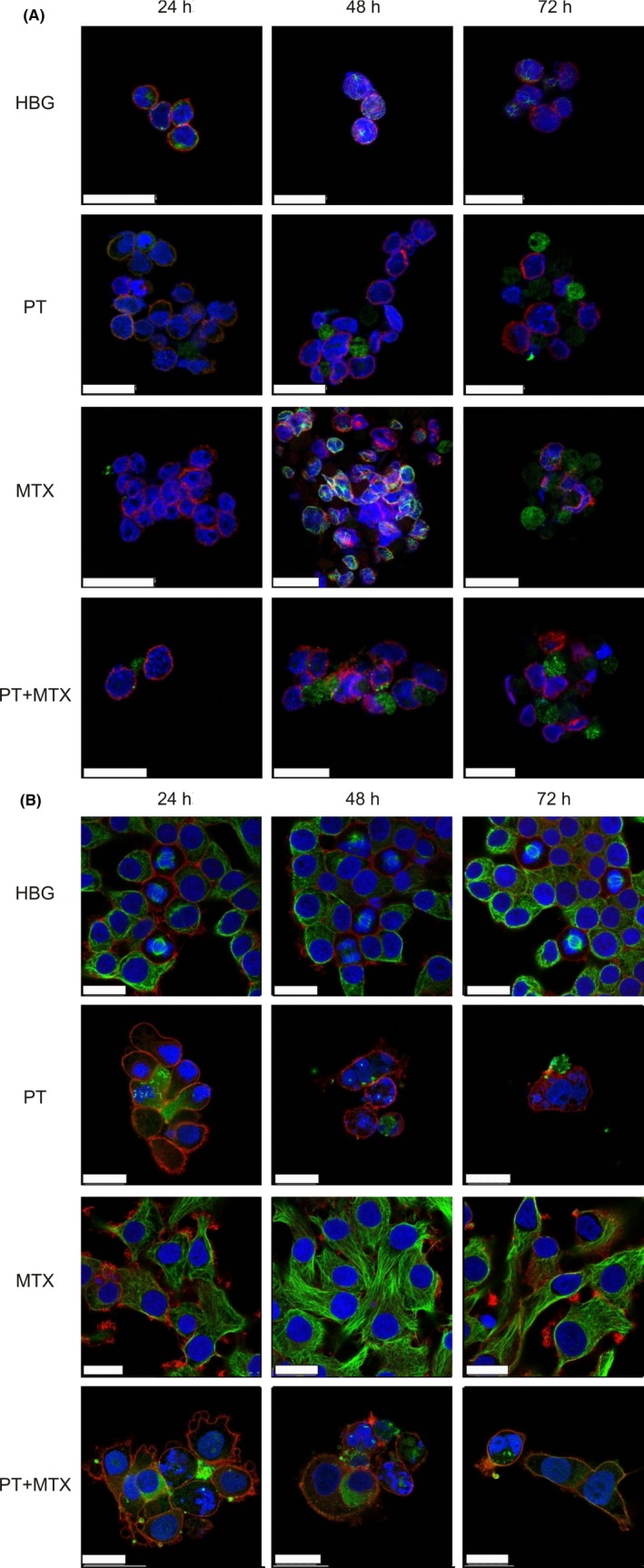
Fluorescence microscopy images of drug‐treated L1210 (A) or KB (B) cells. DNA was stained with DAPI (blue), the F‐actin was stained with phalloidin‐rhodamine (red) and tubulin was visualized using an α‐tubulin primary antibody and an AlexaFluor 488 coupled secondary antibody (green). Pictures show the merged staining, scale bar is 25 μm

The effects of PT, MTX, and PT+MTX on the DNA, the actin cytoskeleton and on the microtubules of L1210 and KB cells were determined by confocal laser scanning microscopy (CLSM). Figure 4 depicts the disruption of the microtubule network of L1210 and KB cells caused by PT treatment, already after 24 hours treatment (see also Figure S5 for nonmerged images). L1210 cells have lost their structural integrity, the microtubule network seems to be located extracellularly. PT also induces nuclear fragmentation of L1210 cell nuclei. Moreover, a change in the F‐actin cytoskeleton upon MTX treatment can be seen especially for KB cells (Figure 4B and Figure S5). Cell morphology changes, as cells become elongated over the 72 hours time course. In comparison to HBG treatment, MTX treatment leads to an accumulation of actin in the cell periphery and the formation of pseudopodia. L1210 cells treated with MTX have completely lost their structural integrity after 72 hours. Furthermore, L1210 cell morphology is changed. Cells are shaped less spherical in comparison to HBG‐treated cells. This effect is, however, less pronounced than for KB cells. PT+MTX‐treated L1210 cells display signs of apoptosis already after 24 hours. Cell integrity is lost, nuclei are fragmented and the microtubule network is destroyed. The toxic effects of the drug combination are equally pronounced on KB cells.

### 3.5 PT+MTX combination treatment in the L1210 leukemia mouse model in vivo

**Figure 5:**
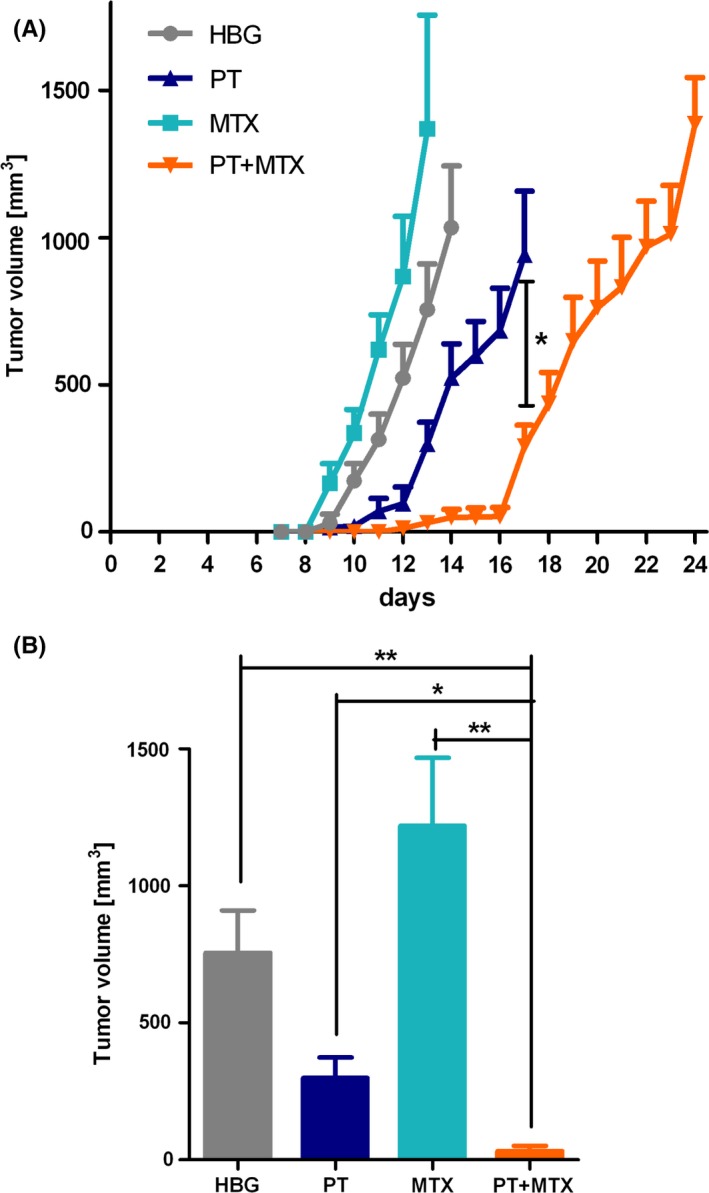
Treatment of subcutaneous L1210 tumors. (A) Tumor volume of subcutaneous L1210 tumors throughout the experiment (mean + SEM; n = 4; *P = 0.0293). Curves end when the first animal is sacrificed, respectively. Animals were treated intravenously with 250 μL of HEPES buffered glucose (HBG), methotrexate (MTX) (5 mg kg^−1^), pretubulysin (PT) (2 mg kg^−1^) or PT+MTX (2 + 5 mg kg^−1^). (B) Comparison of tumor volumes on day 13 after tumor cell inoculation (mean + SEM; n = 4; *P = 0.0132, **P < 0.01)

To further evaluate potential combination effects of PT and MTX, the efficacy of the combinatorial treatment was tested in an in vivo mouse model. After the inoculation of subcutaneous L1210 tumors, mice were randomly divided into four groups (n = 4). Intravenous treatments started on day 3 and were repeated on days 5, 7, 10, 12, 14, 17, and 19 (Figure 5). Animals were sacrificed on day 13 or 14 in case of MTX treatment, and as soon as tumors reached the critical size of 1500 mm^3^ in all other groups. Tumors started to grow around day 9 in the HBG and MTX groups and with a delay of 2 days also in the PT group. In the group treated with PT+MTX, tumor growth could largely be inhibited until day 17, when tumor volume also started to increase (Figure 5A). In a comparison of tumor sizes on day 13 (Figure 5B), tumors of PT+MTX group were significantly smaller than tumors of all other groups (PT+MTX vs PT: *P* = 0.0132). Encouragingly, animals of this group did also survive significantly longer than animals of HBG group (*P* = 0.0062) and mean survival was 5 days longer than in PT‐treated animals. Furthermore, weight development was recorded for the duration of the experiment in order to monitor animal well‐being, especially during injections. Although mice of PT+MTX group gained less weight during the first days, all groups showed a constant weight development throughout the experiment (Figure S6A).

In contrast to the in vitro cytotoxicity, multiple injections of MTX at 5 mg kg^−1^ did not inhibit tumor growth in the L1210 mouse model. Therefore, a new MTX dose finding experiment was performed. Unacceptable cytotoxicity at 100 mg kg^−1^ MTX was observed, since 2 of 4 animals suffered from severe side effects and consequently had to be sacrificed for reasons of animal welfare. However, even under these circumstances or after multiple injections with the highest tolerated dose of 80 mg kg^−1^, we did not achieve a significant growth inhibition of L1210 tumors (Figure S7). It can be excluded that a MTX resistance acquired during the multiple in vivo treatments, since the L1210 tumors after MTX treatment and resection were still MTX sensitive in cell culture (Figure S8).

### 3.6 PT+MTX combination treatment in the human KB carcinoma xenograft mouse model

**Figure 6:**
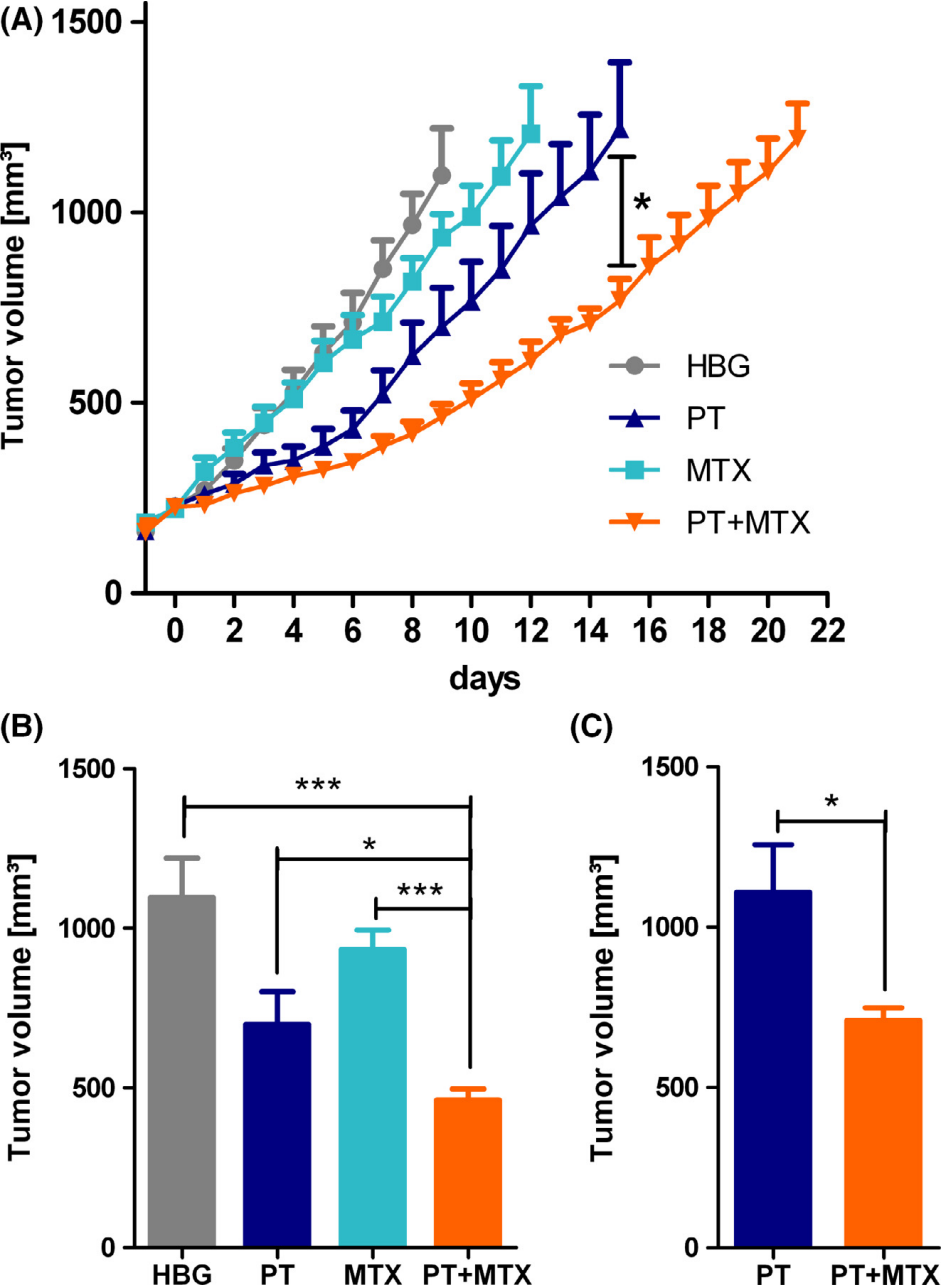
Treatment of subcutaneous KB tumors. (A) Tumor volume (mean + SEM; n = 6 for HEPES buffered glucose (HBG) and methotrexate (MTX), n = 7 for pretubulysin (PT) and combination (PT+MTX) of subcutaneous KB tumors in a xenograft mouse model. Intravenous treatments with 250 μL of HBG buffer control (n = 6), MTX (5 mg kg^−1^, n = 6), PT (2 mg kg^−1^, n = 7) or PT+MTX combination (2 + 5 mg kg^−1^, n = 7) were started individually when tumors reached 200‐250 mm^3^ and were repeated three times per week with a maximum of eight injections. Day‐1 represents 1 day prior to treatment start. Curves end when the first animal is sacrificed, respectively. (B) Comparison of tumor sizes on day 9 after treatment start (mean + SEM; *P < 0.05, ***P < 0.001). (C) Comparison of tumor sizes on day 14 after treatment start (PT vs PT+MTX: *P *=* *0.0230)

Subsequently, the combination therapy was evaluated in the human KB tumor xenograft mouse model (Figure 6). To provide reliable and comparable tumor growth, treatments were started individually as soon as subcutaneous tumors reached 200‐250 mm^3^. Mice were randomly divided into four groups (n = 6 for HBG and MTX, n = 7 for PT and PT+MTX), and treatments with 250 μL of PT, MTX, the PT+MTX combination or HBG were started. Treatments were repeated three times per week with a maximum of eight injections. Weight development and tumor sizes were monitored continuously. Mice were sacrificed when tumor sizes exceeded 1500 mm^3^.

After the treatment start, tumor growth in the HBG‐injected group proceeded rapidly, so the first animal had to be sacrificed after 9 days. Tumor growth in MTX‐treated animals was slightly slowed down after 1 week of treatments and the first animal was sacrificed after 12 days. Notably, tumor growth in both PT containing groups was retarded from the first injection on and could be further inhibited in the PT+MTX combination group (Figure 6A). While the first animal of the PT group had to be sacrificed on day 15 after treatment start, PT+MTX combination led to a survival of all animals until day 21. Figure 6B depicts a comparison of tumor sizes on day 9, indicating that tumors of PT+MTX group are significantly smaller than in all other groups (PT+MTX vs HBG: *P* = 0.0002; PT+MTX vs MTX: *P* < 0.0001; PT+MTX vs PT: *P* = 0.0499). Comparison of tumor sizes on day 14 (Figure 6C) demonstrates a significant antitumoral effect of the drug combination (PT vs PT+MTX: *P* = 0.0230). The weight of animals was monitored regularly; the unobtrusive weight development in all groups indicates that treatments were well tolerated by the animals (Figure S6B).

## 4 DISCUSSION

Drug combinations become increasingly important as standard therapy settings in cancer therapy. While monotherapy approaches often fail,^27^, ^28^ combination therapies represent a promising strategy as they can address the disease from different angles.^29^, ^30^, ^31^

Based on a previous experiment with the novel microtubule inhibitor pretubulysin (PT) conjugated with methotrexate (MTX)‐containing oligomers,^11^ the same drug combination was chosen in this study. By combining PT with the well‐established chemotherapeutic drug MTX, we aimed to further increase the great antitumoral potency of PT. Encouragingly, in vitro cytotoxicity studies demonstrated the favorable combination effect of PT and MTX on both cell lines. The only moderate cytotoxic effects of MTX on KB cells are not surprising, as chemoresistance of KB cells to MTX has previously been reported.^32^, ^33^

Regarding the effects of the drugs on the cell cycle, our experiments confirm the expected G2/M arrest of PT‐treated cells^6^, ^8^, ^34^ and G1/S arrest by MTX.^35^, ^36^ Remarkably, G2/M arrest mediated by PT+MTX combination went far beyond the effect of PT alone. One explanation for the predominance of the PT effect on the cell cycle might be its more direct way of interference. PT binds to tubulin and thereby directly affects the working of the cell division cycle. MTX on the other hand influences the G1/S stage of the cell cycle more indirectly. MTX is essentially a prodrug. In order to bind its target enzyme DHFR, MTX must be polyglutamylated intracellularly. After polyglutamylation, MTX inhibits the synthesis of the co‐factor tetrahydrofolate and thus, the C1 metabolism. As a result, no nucleotides are synthesized which would be essential for DNA synthesis. Furthermore, PT does not have to be converted into its active form before carrying out its toxic effect. The earlier onset in toxicity leads to a G2/M arrest, and arrested cells cannot in turn be affected by MTX anymore.

With regard to drug‐induced apoptotic events in L1210 or KB cells, treatment with PT or MTX alone resulted in a steady increase of apoptotic cells which is in accordance with previous work.^6^, ^10^, ^34^, ^37^ Yet, apoptosis could not be further enhanced by combining both agents.

Previous studies show PT‐induced depolymerization of microtubules in different cell lines,^6^, ^8^ which could be confirmed for L1210 and KB cells using CLSM. Encouragingly, the combination was equally potent as PT alone. Furthermore, we could show the impairment of the cellular actin skeleton mediated by MTX. This is in line with various reports about MTX influence on the actin cytoskeleton.^38^, ^39^ Furthermore, levels and distribution of globular actin (G‐actin) and/or filamentous actin (F‐actin) and total actin were changed upon MTX treatment. Mazur et al^40^ postulate that polyglutamylated MTX inhibits specific enzymes, resulting in increased levels of adenosine. These have in turn been shown to inhibit actin polymerization.^38^, ^41^ It has been demonstrated previously that microtubule inhibitors can influence the actin cytoskeleton.^42^, ^43^, ^44^ Treatment with microtubule depolymerizing drugs increases contractility in fibroblasts. Additionally, rapid restoration of actin‐containing stress fibers is induced, even after their previous disruption.^44^ The authors offer several possible explanations. Firstly, they postulate that cellular forces are redistributed due to the drug‐induced imbalance where the pushing force of microtubules is decreased. This might lead to increased tension and could cause actin to become bundled in stress fibers. Secondly, microtubules may modulate and exert inhibitory control over actin architecture. Microtubules can weaken contractility and organization of actin. Microtubule disruption releases actin from inhibition.^44^ As to a PT+MTX combination effect, one may hypothesize that actin stress fibers might be required in the cellular survival response upon microtubule inhibition by PT. MTX treatment has been shown to prevent such actin stress fibers. Thus, in the PT+MTX combination, one can assume that the cell rescue effect by actin is prevented by MTX, leading to a combined loss in microtubule as well as actin cytoskeleton function and thus enhanced cell killing. In addition to the qualitative analysis of CLSM, more quantitative experiments will be required to further analyze the underlying molecular mechanisms.

In contrast to cell culture cytotoxicity, a lack of antitumoral activity of MTX was observed in vivo at the applied dosage of 5 mg kg^−1^, which is supported by previous studies.^45^, ^46^ The dosage of MTX used in the mouse experiments was based on efficacy in cell culture. Burger et al described 100 mg kg^−1^ as the maximum tolerated dose of MTX for NMRI‐nude mice.^47^ In our additional MTX dose finding experiments, even after multiple injections with the highest tolerated dose of 80 mg kg^−1^, we did not observe any significant L1210 tumor growth inhibition in vivo. An acquired chemoresistance against MTX can be excluded, since the MTX‐treated tumors are still MTX sensitive in cell culture. On the other hand, PT at the well‐tolerated 2 mg kg^−1^ dose exhibited a clear antitumoral effect on L1210 tumors in vivo. This is also in accordance with our recent work in combining PT with antitumoral EG5 siRNA.^9^ Encouragingly, this favorable PT effect could be further enhanced by the co‐administration of the rather low dose of 5 mg kg^−1^ MTX, resulting in a significantly retarded tumor growth in the combination group. The boosting effect of low dose MTX is remarkable, considering the lack of antitumoral effects of the single drug at even 20‐fold higher dose.

In the KB human cervix carcinoma tumor model, PT had already previously demonstrated antitumoral effects.^11^ In this study, the antitumoral activity of 2 mg kg^−1^ PT could be confirmed, while MTX only slightly inhibited KB tumor growth. This is consistent with the known in vitro chemoresistance of KB cells to MTX^48^ and in vivo studies.^46^ Importantly, also in this carcinoma model, the co‐administration of 5 mg kg^−1^ MTX resulted in a significantly enhanced antitumoral effect of PT. Very similar findings were made in a HUH7 hepatocellular carcinoma model in NMRI‐nude mice, with MTX showing negligible effects, whereas both PT containing groups exhibited significantly inhibited tumor growth and survival of all animals for 22 days for PT vs 25 days for PT+MTX (SK, veterinary doctoral thesis, LMU 2019).

Relevant for our work, the combination of MTX with Vinca alkaloids as another class of tubulin binders has been demonstrated as favorable for cancer therapy both in a leukemia mouse model^49^ and in the clinics.^50^ Chello et al observed a beneficial combination effect of vincristine with MTX in vitro and in vivo. In a study with patients suffering from aggressive recurrent fibromatosis, Park et al demonstrated the combination of vinblastine with low dose MTX to be effective and well tolerated. Thus, the combination of the novel potent tubulin inhibitor PT with MTX might present a new interesting clinical direction in cancer chemotherapy.

## DISCLOSURES

None declared.

## AUTHOR CONTRIBUTIONS

Participated in research design: S. K., I. T., E. W., A. M. V., S. Z.; Conducted experiments: *S*. K., I. T., M. H.; Contributed new reagents or analytic tools: J. G., U. K.; Performed data analysis: S. K., I. T., E. W., A. M. V., S. Z.; Wrote or contributed to the writing of the manuscript: S. K., I. T., E. W., S. Z.
