# Supplementary material for: Combined antitumoral effects of pretubulysin and methotrexate
Source: Pharmacol Res Perspect. 2019 Jan 22;7(1):e00460. doi: 10.1002/prp2.460 (PMC6343018; doi:10.1002/prp2.460)
Supplement: Supplementary file 1 [file PRP2-7-e00460-s001.docx]

**Supporting Information**

For the paper

**Combined Antitumoral Effects of Pretubulysin and Methotrexate**

Sarah Kern, Ines Truebenbach, Miriam Höhn, Jan Gorges, Uli Kazmaier, Stefan Zahler, Angelika M. Vollmar, Ernst Wagner

**Table of Content**

1. ***In vitro* experiments**
   1. Dose ratio evaluation
   2. MTT assay in 12-well plates
   3. Apoptosis analysis
   4. Confocal laser scanning microscopy images
2. ***In vivo* experiments**

2.1 PT dose finding

2.2 PT+MTX combination treatment experiment in L1210 and KB tumor model

2.3 MTX dose finding in L1210 tumor model

2.4 MTT assay of *in vivo* passaged L1210 cells

1. **Summary of findings**
2. **References**

**1. *In vitro* experiments**

**1.1 Dose ratio evaluation**

Different molar ratios of MTX to PT were investigated in this study. The PT dose was left constant while the MTX dose was gradually increased from equimolar to a 10‑fold molar surplus of MTX to PT. **Figure S1A** depicts the MTT data of drug treated L1210 cells at the different ratios. As expected, an increase in the MTX dose leads to a reduction in cell viability of the MTX sensitive L1210 cells when looking at the graphs from left to right. For all dose ratios, the combination of PT+MTX exceeds PT and MTX in terms of cell killing.

Cell viability after drug treatment of KB cells with different molar ratios of PT, MTX or PT+MTX is shown in **Figure S1B**. Also here, a steady increase of MTX leads to an increase of the number of dead cells. The cell viability of MTX resistant KB cells, however, cannot be reduced to less than 40% cell viability, even at the highest dose of 1 µM of MTX. At the low molar ratios (1 to 1 and 1 to 3) the combination seems to be slightly superior to the single drugs. That effect cannot be observed for the higher MTX concentrations at the ratios 1 to 5 and 1 to 10 though.

The dose ratio of 1 to 3 was chosen for all further experiments due to a previously performed treatment study where said ratio led to a promising retardation in tumor growth (Truebenbach et al., 2017).


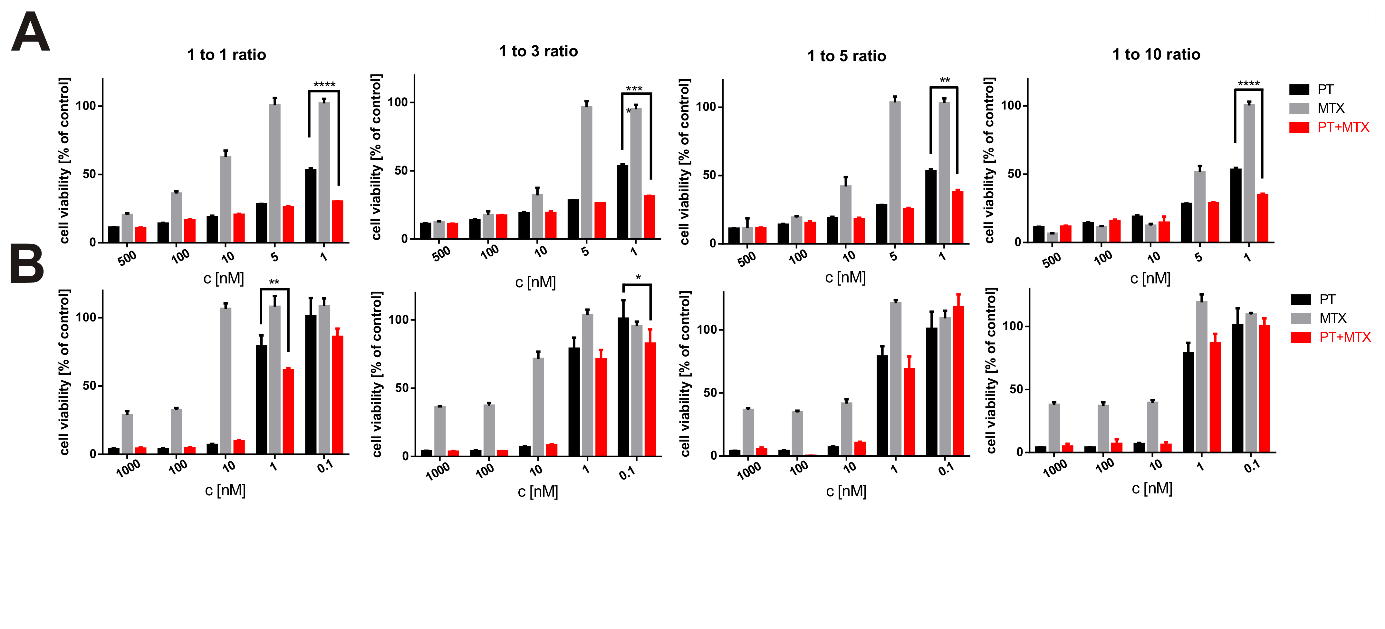
**Figure S1 Evaluation of different dose ratios of MTX to PT by MTT assay.** Cell viability of drug treated **A** L1210 and **B** KB cells. Cells were treated with PT, MTX and the combination PT+MTX at different molar ratios. Cell viability was measured with an MTT assay after 72 h incubation time and is presented as the mean + SD (n = 5) in % relative to HBG treated cells.

**1.2 MTT assay in 12-well plates**

Different conditions were used to determine the *in vitro* effects of PT, MTX and PT+MTX. The MTT assays to determine IC50 values of drugs and their possible combination effects were performed in 96‑well plates in accordance to previous work on cellular effects of PT, with drug concentrations ranging from 0.0001 nM up to 100 nM of PT (Truebenbach et al., 2017, Braig et al., 2014, Kubisch et al., 2014).

For flow cytometry experiments, higher numbers of cells were needed. Hence, experimental conditions were modified. Cells were seeded in 12-well plates and suitable concentrations of PT and MTX were determined. The effect of 200 nM PT and 600 nM MTX on L1210 and KB cells were determined by MTT-assay.

**Figure S2A** depicts the time-dependent effects of drugs on L1210 cells. As expected, cell viability decreases over the time course of the experiment, with only 11% viable L1210 cells left after 72 h of PT+MTX treatment, 16% viable cells after PT treatment and 17% viable cells after MTX treatment. The antitumoral effect of PT is already well visible after 24 h, whereas the onset of MTX toxicity can be seen after 48 h. The combination effect of PT+MTX over PT is already prominent after 24 h treatment.

The weaker overall effect of the drugs on KB cells can be seen in **Figure S2B**. After 72 h incubation, cell viability is reduced to 22% (PT+MTX), 20% (PT) or 57% (MTX). No significant combination effect is visible in this setting. A time-dependent effect of drugs on cell viability is also visible here.

**
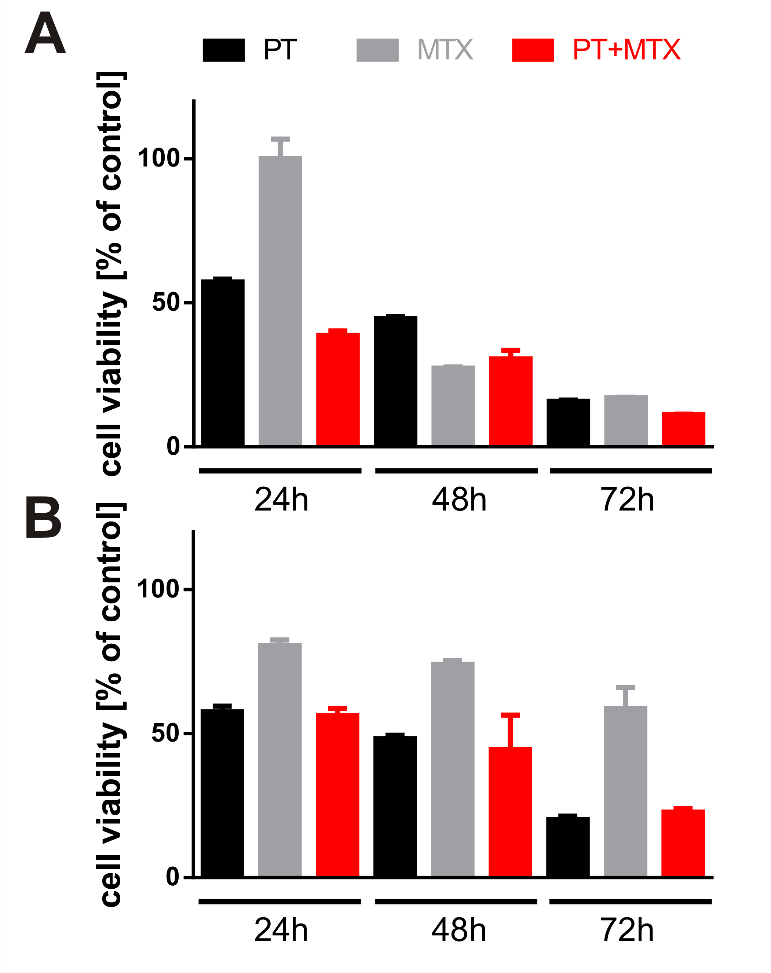
**

**Figure S2 MTT assay of drug treated L1210 (A) and KB cells (B)** at 24, 48 and 72 h incubation time. Cell viability is presented as mean + SD (n = 5) in % relative to buffer (HBG) treated cells.

**1.3 Apoptosis analysis**

**
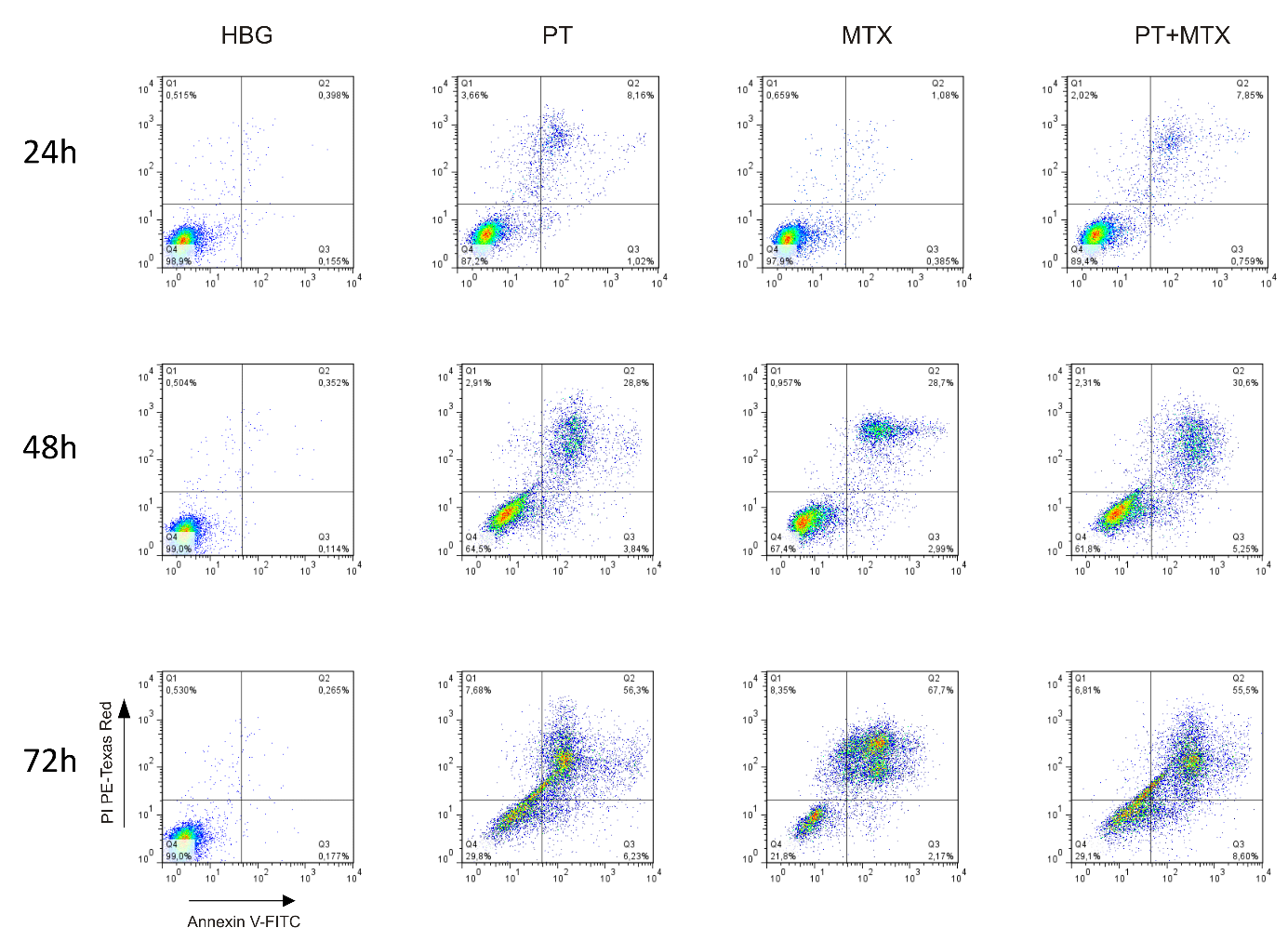
**

**Figure S3 Flow cytometric analysis of apoptosis in drug treated L1210 cells.** L1210 cells were treated with HBG, PT, MTX and PT+MTX and incubated for 24, 48 and 72 h respectively. Cells were stained with annexin V-FITC and propidium iodide and analyzed by flow cytometry. Treatments were performed in triplicates (n = 3).

Q1: FITC – / PI + Q2: FITC +/PI + Q3: FITC +/PI – Q4: FITC –/PI –


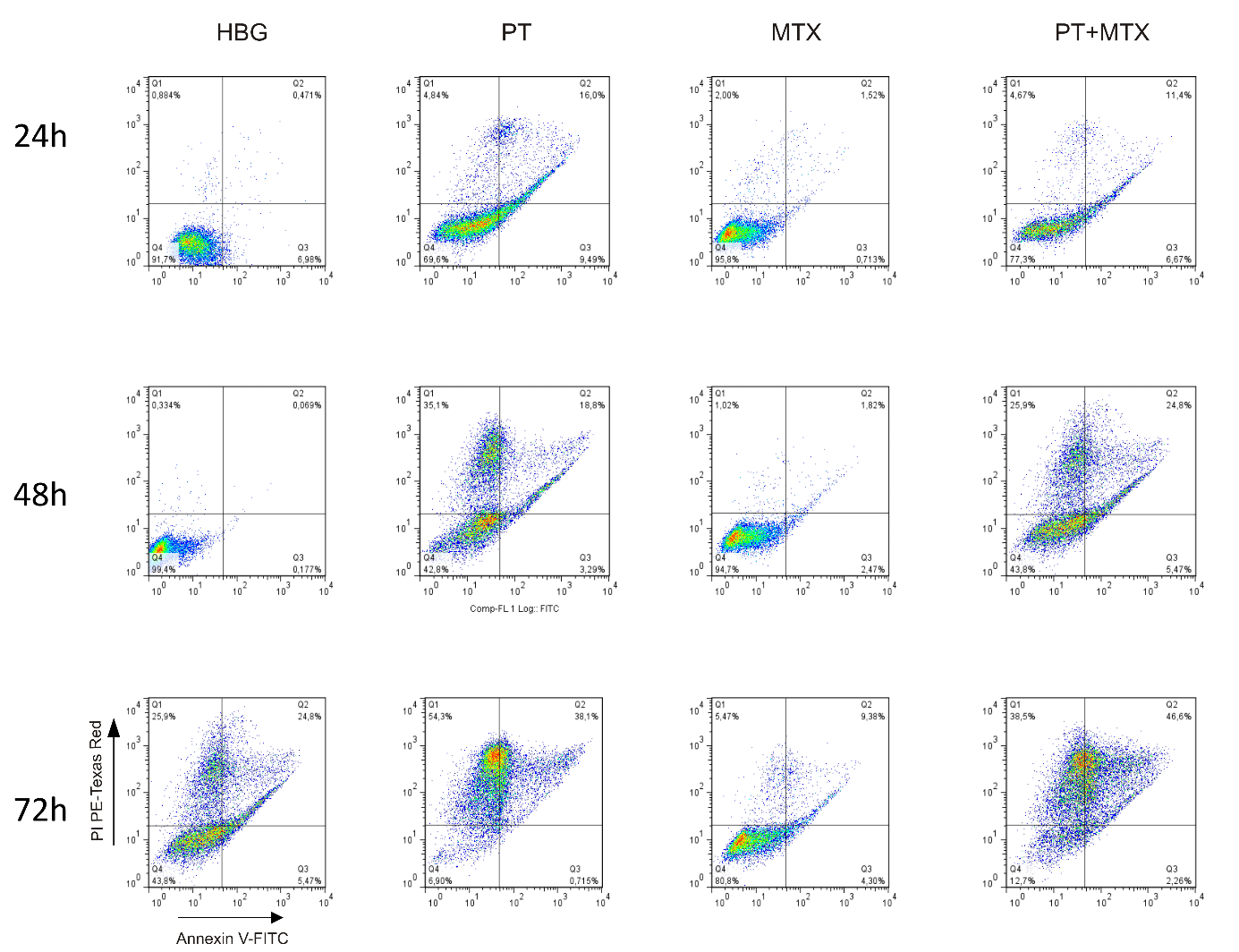


**Figure S4 Flow cytometric analysis of apoptosis in drug treated KB cells.** KB cells were treated with HBG, PT, MTX or PT+MTX and incubated for 24, 48 and 72 h respectively. Cells were stained with annexin V-FITC and propidium iodide and analyzed by flow cytometry. Treatments were performed in triplicates (n = 3).

Q1: FITC – / PI + Q2: FITC +/PI + Q3: FITC +/PI – Q4: FITC –/PI –

- 1. **Confocal laser scanning microscopy images of drug treated cells**

**
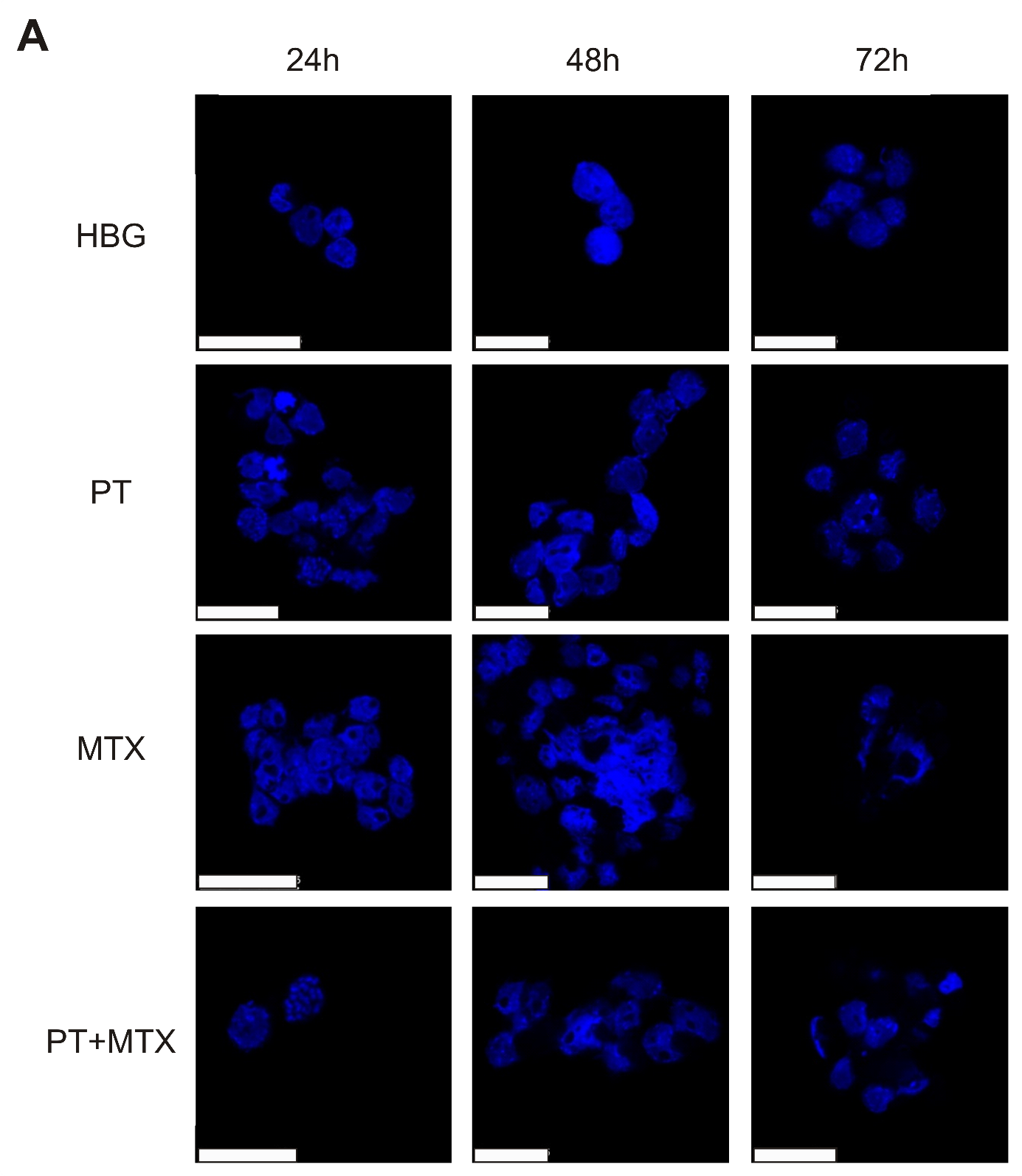
**

**
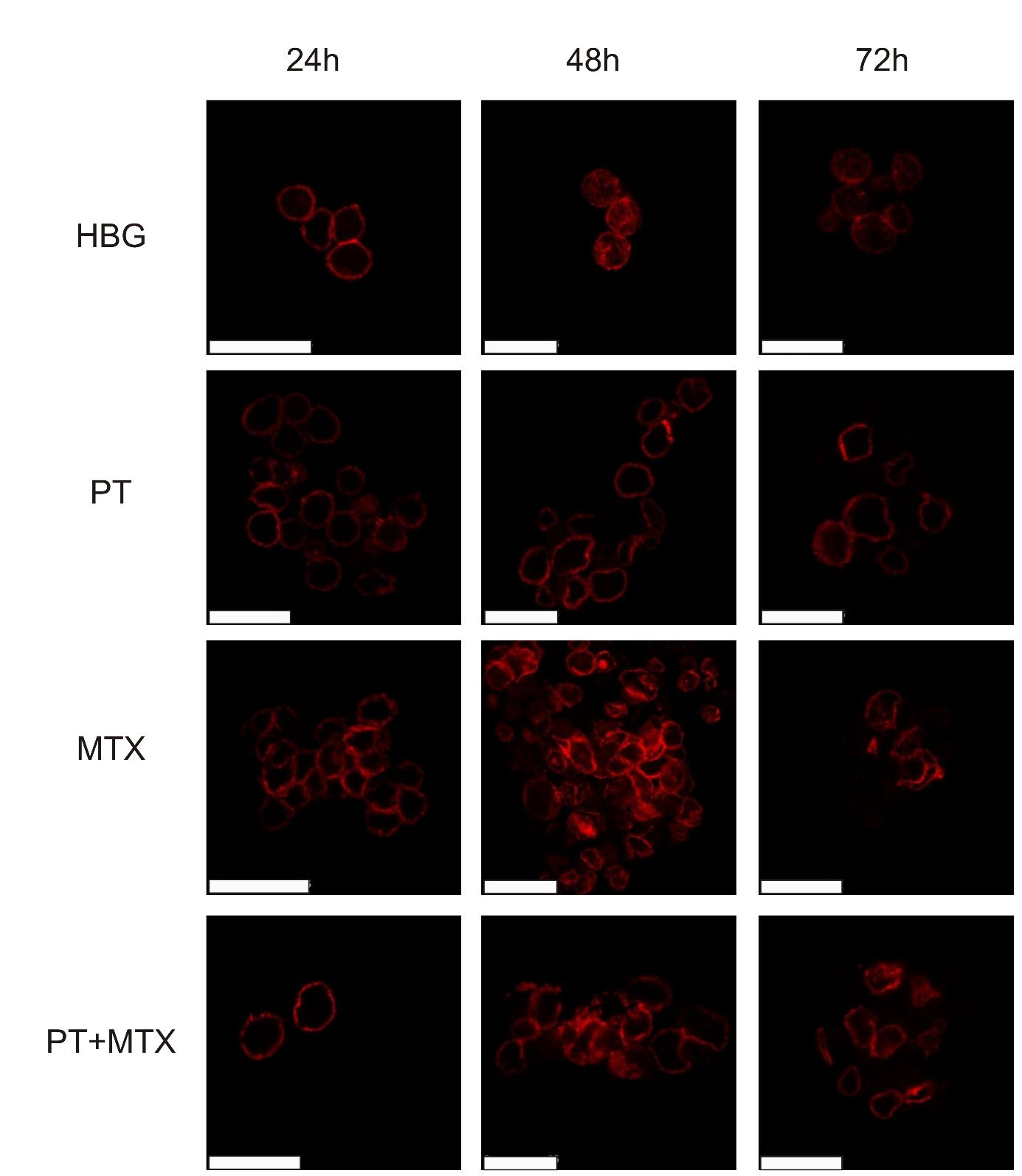

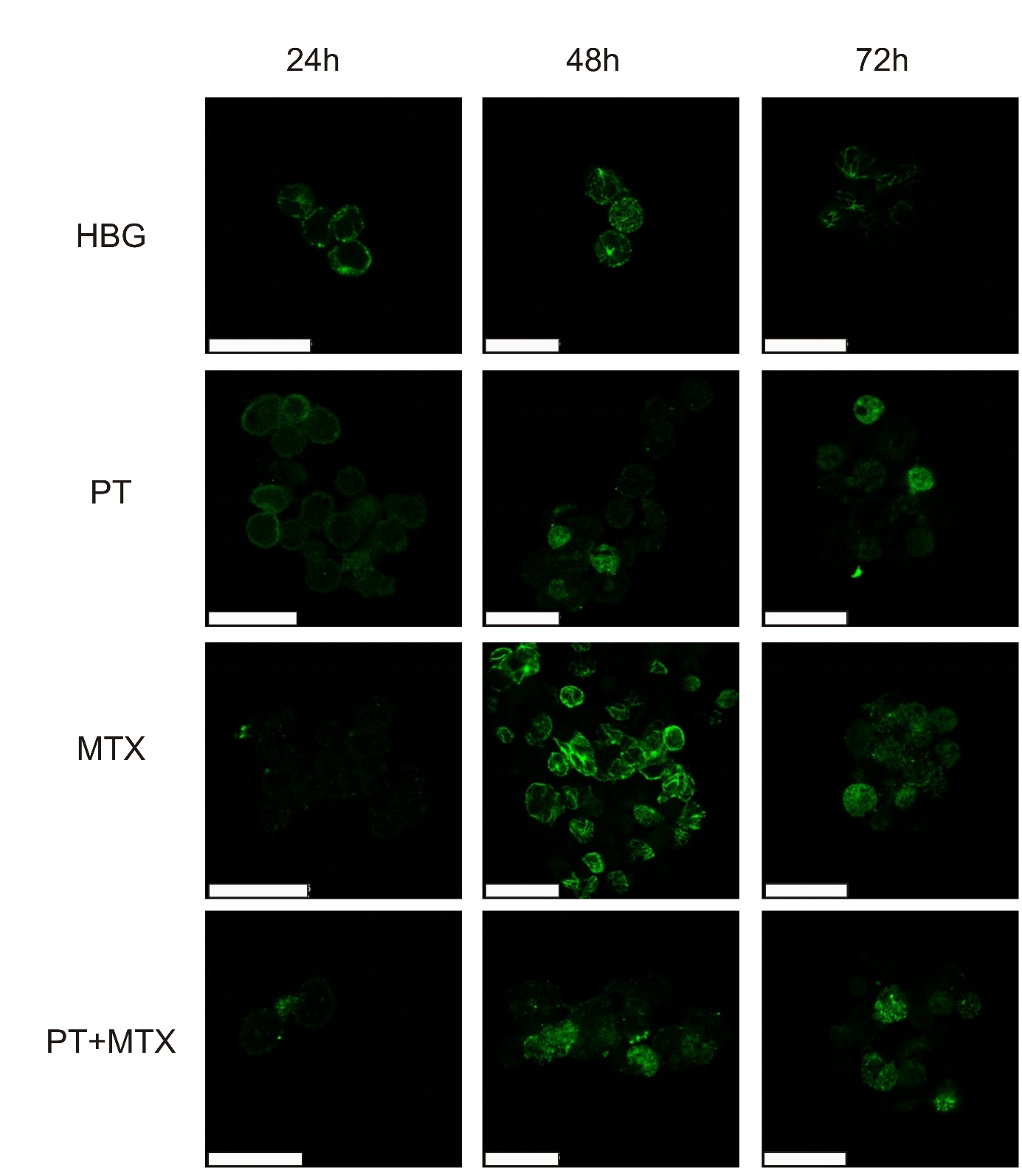
**

**
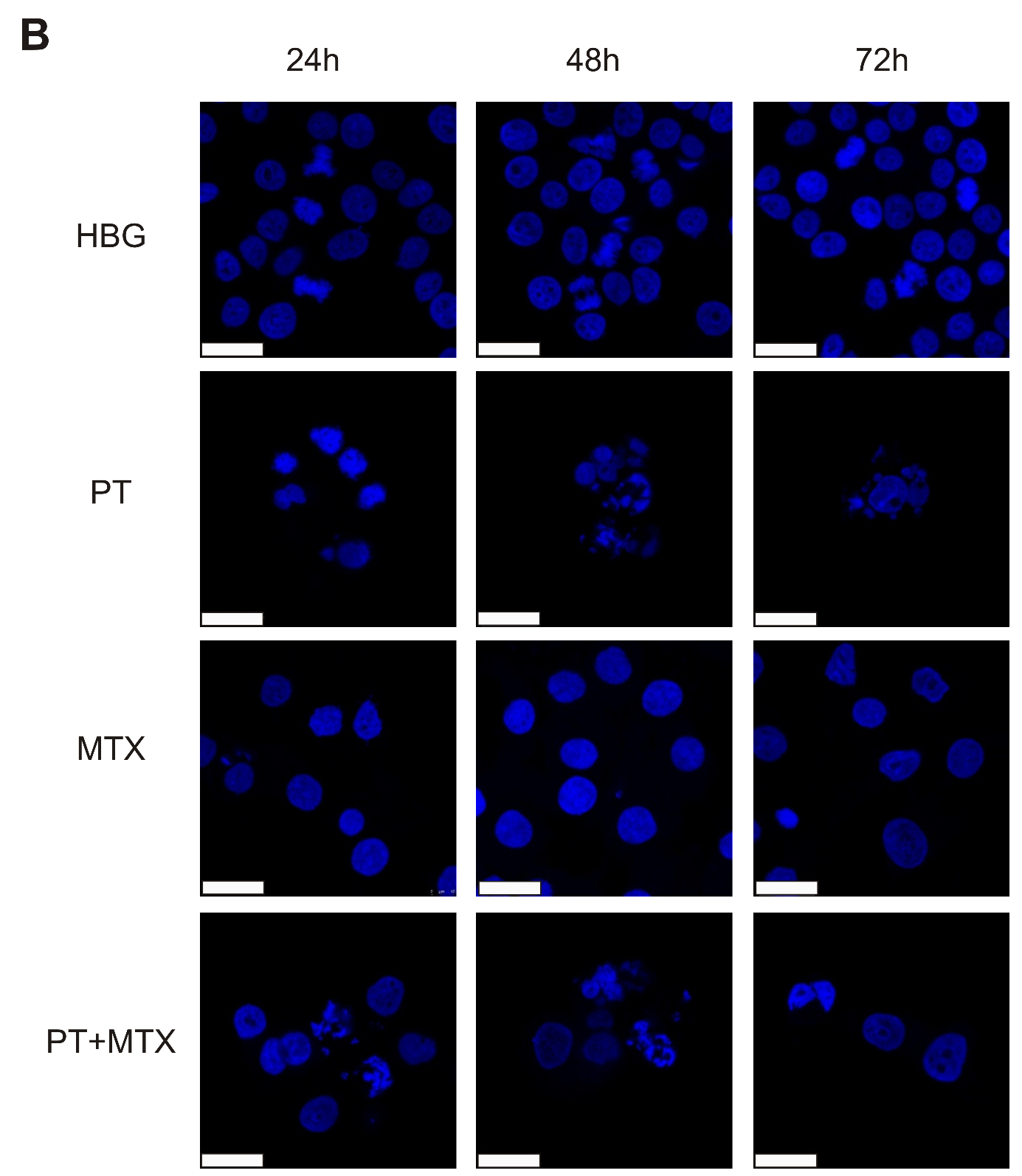

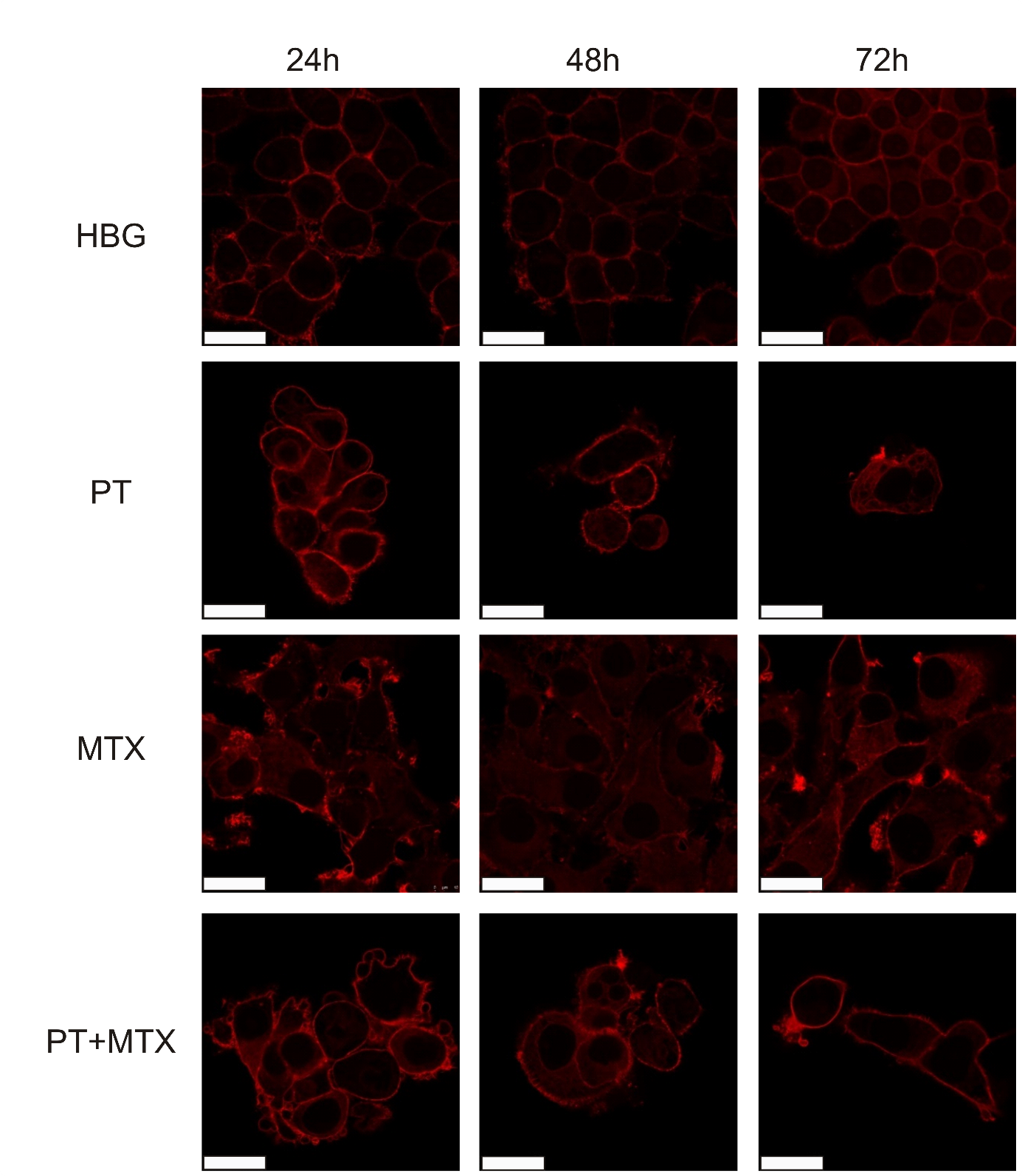

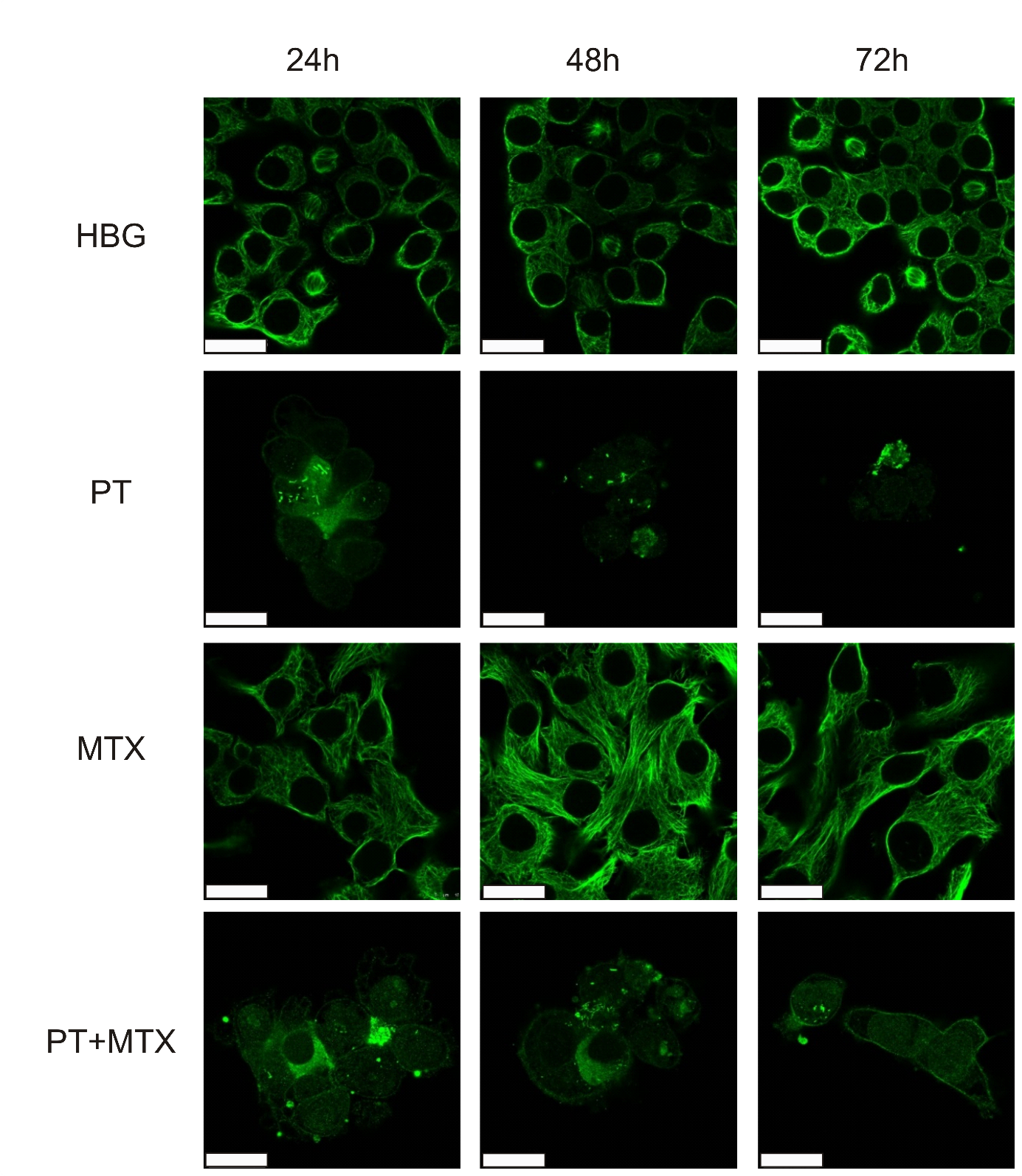
**

**Figure S5 Confocal laser scanning microscopy images of drug treated L1210 (A) and KB cells (B).** DNA was stained with DAPI (blue), tubulin was visualized using an α-tubulin primary antibody and an AlexaFluor 488 coupled secondary antibody (green), the actin cytoskeleton was stained with phalloidin-rhodamine (red).

**2. *In vivo* experiments**

**2.1 PT dose finding**

In order to determine an optimum dose of PT, we performed an internal dose finding experiment based on doses used in successful *in vivo* experiments published by other groups (Braig et al., 2014, Rath et al., 2012, Kretzschmann et al., 2014). KB tumor bearing animals were injected every second day with 0.1, 0.2 or 0.4 mg/kg of PT. However, we did not find any advantageous KB tumor growth inhibition after these multiple intravenous injections. Subsequently, two single animals were injected with 1.5 mg/kg respectively 3 mg/kg PT. While the former dose was well accepted, the animal injected with the latter dose showed side effects in the form of severe weight loss after the 5^th^ treatment and had to be sacrificed for reasons of animal welfare. We later on performed a combination treatment experiment with 8 intravenous injections of PT at a dose of 2 mg/kg and achieved effective tumor growth inhibition in the absence of any systemic side effects (Klein et al., 2018). Therefore, PT+MTX experiments of the current study were carried out with the same dose.

**2.2 PT+MTX combination treatment experiment in L1210 and KB tumor model**

**
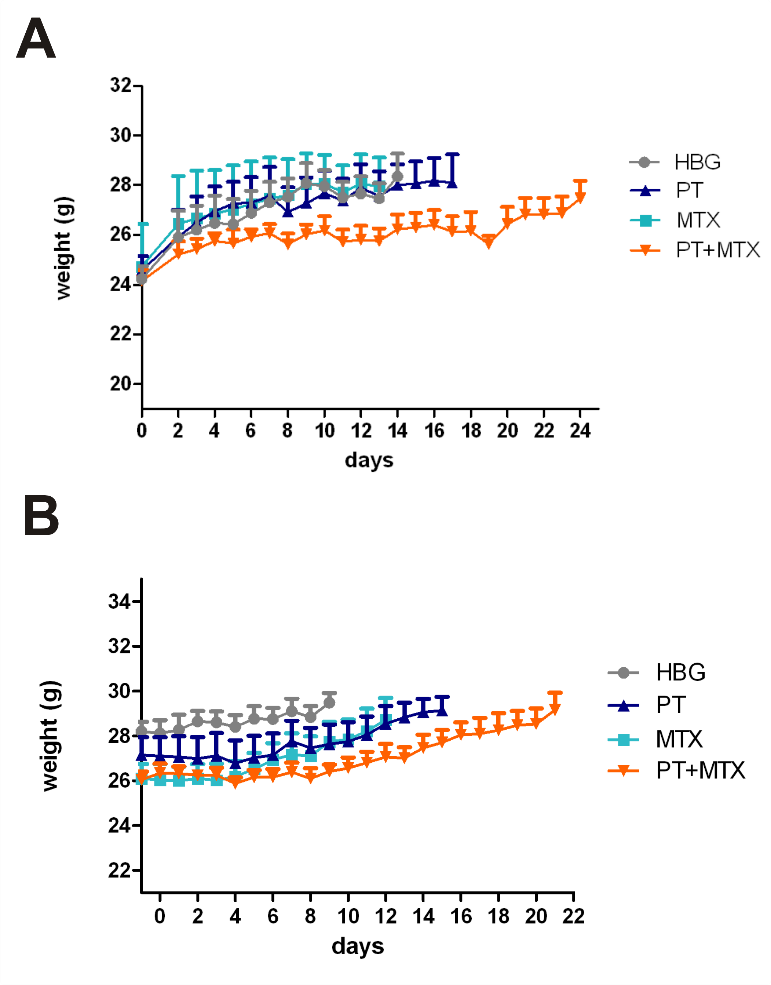
**

**Figure S6 Weight development throughout PT+MTX combination therapy experiment. A** in L1210 tumor bearing animals, starting on day 0 with tumor cell inoculation (mean + SEM, n = 4) and **B** in KB tumor bearing animals, starting on day -1, one day prior to treatment start (mean + SEM, n = 6 for HBG and MTX, n = 7 for PT and PT+MTX). Curves end when the first animal is sacrificed, respectively.

**2.3 MTX dose finding in L1210 mouse tumor model**

**Method:**

MTX dose finding was performed in 2 parts under equal conditions. Animals were injected subcutaneously with 0.5 × 10⁶ L1210 cells in 150 µL PBS and randomly distributed into 4 respectively 6 groups. Treatments were started on day 3 and repeated 3 times per week with a maximum of 7 injections. In the first part, low doses of MTX were evaluated for their effect on tumor growth. Therefore, mice were treated with 2.5, 5, 7, 10 or 20 mg/kg MTX. In the second section, higher doses of 40, 80 and 100 mg/kg MTX were administered. Mice were sacrificed by cervical dislocation once their tumor reached 1500 mm³ or in case of severely affected well-being.

**Results:**

To evaluate the best working dose for tumor growth inhibition *in vivo*, mice were inoculated with L1210 cells and injections of MTX respectively HBG were carried out three times per week. Neither in the first part of the experiment comparing lower doses of 2.5, 5, 7, 10 and 20 mg/kg nor in the second part comparing 40, 80 and 100 mg/kg we could observe any significant antitumoral effect of MTX as compared to HBG treated animals (**Figure S7A+B**). However, especially in the group treated with the highest dose of 100 mg/kg, side effects in the form of severe weight loss and notably affected well-being occurred (**Figure S7C**). Consequently, 2 out of 4 animals had to be sacrificed before reaching the critical tumor volume for reasons of animal welfare.


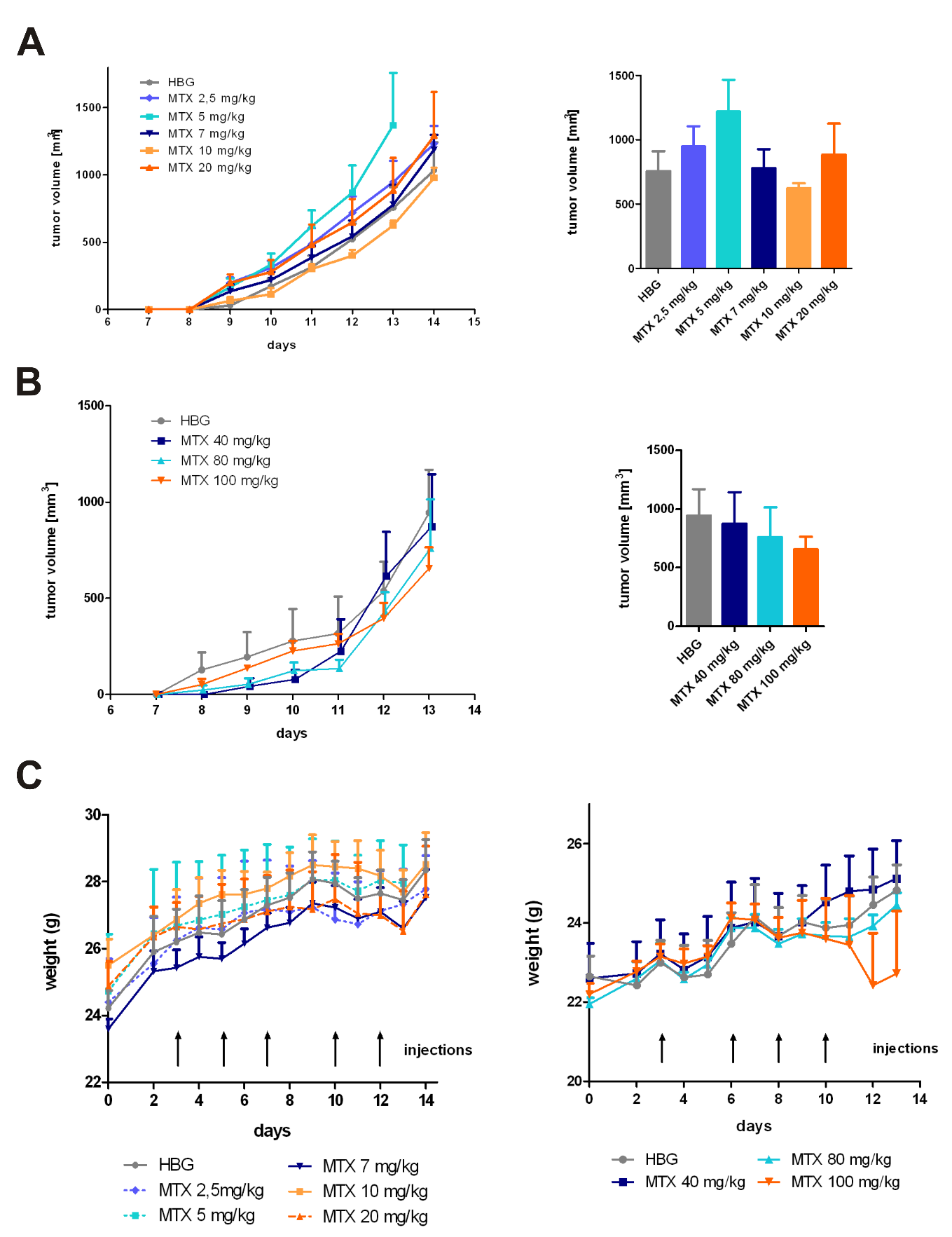


**CV**

**Figure S7 MTX dose finding experiments in L1210 tumor mouse model. A** Tumor growth and comparison of tumor sizes on day 13 of animals treated with doses of 2.5, 5, 7, 10 and 20 mg/kg of MTX. **B** Tumor growth and comparison of tumor sizes on day 13 of animals treated with 40, 80 and 100 mg/kg of MTX. **C** Weight development of animals in both experimental parts. Intravenous injections are indicated by black arrows (mean + SEM; n = 4). Curves end when the first animal is sacrificed, respectively.

**2.4 MTT assay of *in vivo* passaged L1210 cells**

Due to the discrepancies in the *in vitro* and *in vivo* MTX sensitivity of L1210 cells, *in vivo* passaged L1210 cells were investigated for a possible MTX resistance formation that can develop after multiple drug treatments. Therefore, tumors were removed after euthanasia of one mouse of the 5 mg/kg and 80 mg/kg treatment group respectively. The tumors were shredded into small pieces to extract the cells. The mass of tissue and cells was transferred into a membrane filter and centrifuged for 5 min at 1000 rpm to remove tissue and gain the cells in the filtrate. The cell containing filtrate was cultured in a standard cell culture flask under abovementioned conditions. Cells were seeded after passaging them once *in vitro* and an MTT assay was performed. **Figure S8** depicts cell viability of *in vivo* passaged cells that were treated with MTX for 72 h. Also, at a high *in vivo* dose of 80 mg/kg, no resistance of L1210 cells to MTX could be observed.


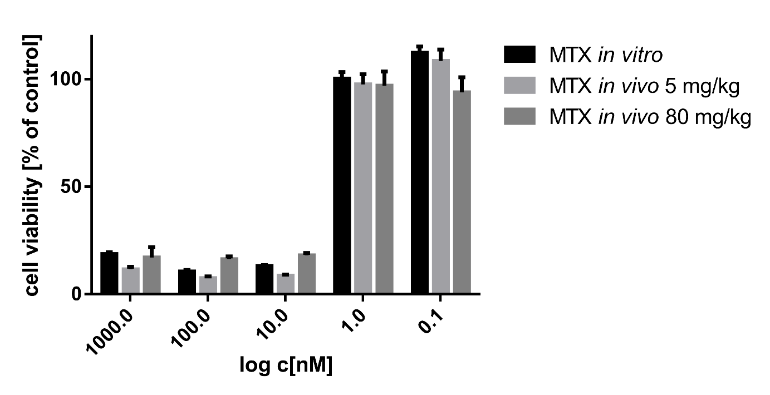


**Figure S8 MTT assay of *in vivo* passaged L1210 cells** of mice treated with either 5 mg/kg MTX or 80 mg/kg MTX. Cell viability after 72 h incubation time. Cell viability is presented as mean + SD (n = 5) in % relative to buffer (HBG) treated cells.

**3. Summary of findings**

|  | L1210 cells | KB cells |
| --- | --- | --- |
| ***In vitro*** |  |  |
| MTT data (cytotoxicity) | PT: +  MTX: +  Combination: **synergism** | PT: +  MTX: (-) resistance  no combination effect |
| Cell cycle | PT: G2/M arrest  MTX: G1/S arrest  Combination: **strong G2/M** arrest | PT: moderate G2/M arrest  MTX: weak G1/S arrest  Combination: **strong G2/M** arrest |
| Apoptosis | PT: +  MTX: +  no combination effect | PT: +  MTX: (-) resistance  no combination effect |
| Cytoskeleton | PT: microtubule disruption, nuclear fragmentation +  MTX: effect on actin skeleton & cell morphology +  **combination effect +** | PT: microtubule disruption, nuclear fragmentation +  MTX: effect on actin skeleton & cell morphology ++  **combination effect +** |
| ***In vivo*** |  |  |
| Tumor growth inhibition | PT: +  MTX: -  **Combination: +** (p = 0.0132) | PT: +  MTX: -  **Combination: +** (p = 0.0230) |
| Prolonged survival | PT: +  MTX: -  **Combination: +** (p = 0.3549) | PT: +  MTX: -  **Combination: +** (p = 0.4686) |

**4. References**

Braig S, Wiedmann RM, Liebl J, Singer M, Kubisch R, Schreiner L, Abhari BA, Wagner E, Kazmaier U, Fulda S, and Vollmar AM (2014) Pretubulysin: a new option for the treatment of metastatic cancer. *Cell Death Dis,* 5**,** e1001.

Klein PM, Kern S, Lee DJ, Schmaus J, Höhn M, Gorges J, Kazmaier U, and Wagner E (2018) Folate receptor-directed orthogonal click-functionalization of siRNA lipopolyplexes for tumor cell killing in vivo. *Biomaterials*, 178, 630-642.

Kretzschmann VK, Gellrich D, Ullrich A, Zahler S, Vollmar AM, Kazmaier U, and Fürst R (2014) Novel tubulin antagonist pretubulysin displays antivascular properties in vitro and in vivo. *Arterioscler Thromb Vasc Biol,* 34**,** 294-303.

Kubisch R, Von Gamm M, Braig S, Ullrich A, Burkhart JL, Colling L, Hermann J, Scherer O, Müller R, Werz O, Kazmaier U, and Vollmar AM (2014) Simplified pretubulysin derivatives and their biological effects on cancer cells. *J Nat Prod,* 77**,** 536-42.

Rath S, Liebl J, Fürst R, Ullrich A, Burkhart JL, Kazmaier U, Herrmann J, Müller R, Gunther M, Schreiner L, Wagner E, Vollmar AM, and Zahler S (2012) Anti-angiogenic effects of the tubulysin precursor pretubulysin and of simplified pretubulysin derivatives. *Br J Pharmacol,* 167**,** 1048-61.

Truebenbach I, Gorges J, Kuhn J, Kern S, Baratti E, Kazmaier U, Wagner E, and Lächelt U (2017) Sequence-Defined Oligoamide Drug Conjugates of Pretubulysin and Methotrexate for Folate Receptor Targeted Cancer Therapy. *Macromol Biosci,* 17, 1600520.
